# Alternative green application areas for olive pomace catalytic pyrolysis biochar obtained via marble sludge catalyst

**DOI:** 10.1007/s10532-024-10088-z

**Published:** 2024-07-01

**Authors:** Gamze Goktepeli, Afra Ozgan, Vildan Onen, Gulnare Ahmetli, Merve Kalem, Esra Yel

**Affiliations:** 1 Environmental Engineering Department, Engineering and Natural Sciences Faculty, Konya Technical University, Konya, Turkey; 2 Mining Engineering Department, Engineering and Natural Sciences Faculty, Konya Technical University, Konya, Turkey; 3 Chemical Engineering Department, Engineering and Natural Sciences Faculty, Konya Technical University, Konya, Turkey

**Keywords:** Catalytic pyrolysis, Biomass to energy, Marble sludge, Olive pomace, Olive pomace biochar

## Abstract

Evaluating industrial wastes in the system with minimum preprocessing and generation economically valuable products from them have critical importance. In this regard, especially cheap, wieldy, and readily available catalysts have been researched to increase variety of useful products in pyrolysis systems, to reduce process time, and to increase quality and diversity of products. Therefore, in this study, marble sludge (named K1) was evaluated as catalyst at different dosages (10%, 20%, 30%, 50%) and pyrolysis temperatures (300, 500, 700 °C) in olive pomace (OP) pyrolysis and; the potential green applications of produced new biochars at new usage areas with different purposes based on characteristics were investigated. ANOVA test results showed that temperature and catalysts ratio had significant effect on pyrolysis product yields since significance value for K1 and temperature was lower than 0.05 for pyrolysis products. OP-K1 biochars had alkaline properties and high earth metal quantities. Moreover, increment in K1 ratio and temperature resulted in decrement of the biochar surface acidity. Therefore, it can be indicated that these biochars can have a potential usage for anaerobic digestion processes, lithium-ion batteries, and direct carbon solid oxide fuel cell (DC-SOFC) but further electrochemical property test should be performed. Moreover, produced biochars can be alternative fuels in some processes instead of coal since they have low S content and high heat values. Consequently, it is foreseen that produced biochars will have an important place in the development of potential usage areas with a new and environmentally friendly approach in different areas apart from the conventional uses of catalytic pyrolysis chars.

**Graphical abstract:**

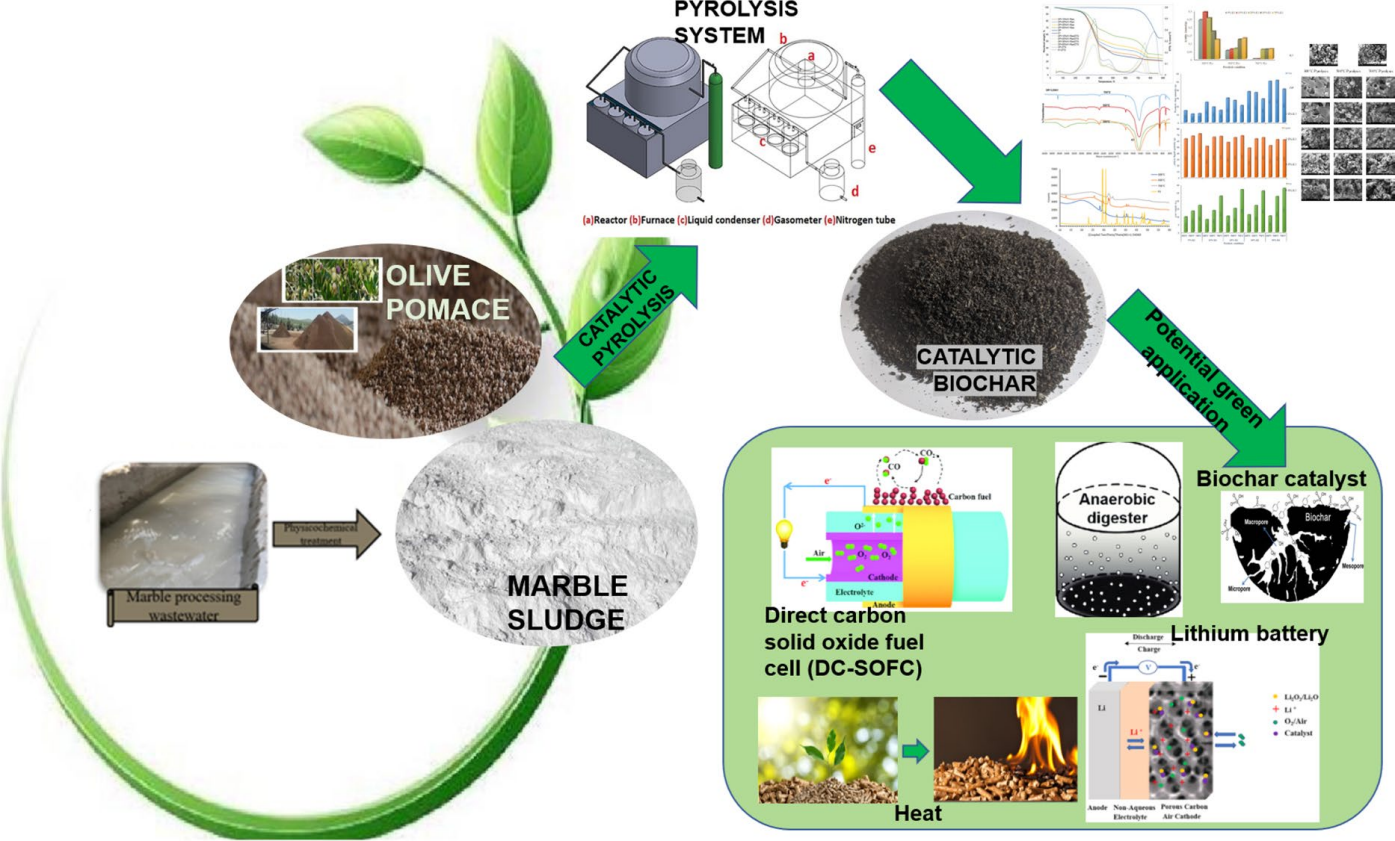

## Introduction

Thermochemical processes, such as pyrolysis, can be classified in the impressive methods to convert biomass into both fuel and high added-value products. Biomass usage for energy generation either directly or indirectly, has becoming an attractive topic due to several advantages as they are widely available and they are periodically generated in high production amounts (Tawalbeh et al. 2021). Therefore, they are considered as sustainable resources. For instance, nearly 1700 billion tons of biomass is generated in one year which means 600 billion tons of oil or 850 billion tons of standard coal (Liang et al. 2021). Pyrolysis occurs at high temperature in oxygen-free environment and biochar, pyrolysis liquid and gas fractions are generated at the end of the biomass pyrolysis. Pyrolysis biochar products consist of mainly fixed carbon, volatile substances, moisture, and a small amount of ash. Pyrolysis biochars have been used commonly in the land applications for many years due to its high stability and long-term carbon sinks for the soil. However, nowadays new green approaches for biochar potential uses, especially in electrochemical cells, are becoming more common. Batteries and supercapacitors’ performance is highly depended on the carbonaceous material. Activated charcoal, carbon nanotubes and graphene can be used in the supercapacitor production. Another alternative for the usage of biochars is as anode of a lithium battery. For instance, Chen et al. (2021) reported that when biochar of *Eichhornia crassipes* which is rich in the in heteroatoms (N, O etc.) was used as anodes for lithium-ion batteries, they obtained high initial reversible specific capacity. Similarly, Kane et al. (2022) emphasized that 2.4 kg CO_2_ eq greenhouse gas emission in the lithium-ion batteries by petroleum-derived additive can be decreased to the 1.2 kg CO_2_ eq by using renewable biochar additive. Another new usage area for the biochars is direct carbon solid oxide fuel cell (DC-SOFC) which are the green-efficient conversion device of the carbon fuel to the electricity directly. Xie et al. (2021) reported that pure walnut shell biochar can be used effectively as a fuel in DS-SOFC. Addition to all these, biochars also can be utilized in energy applications since char is a type of charcoal with high energy content. In pyrolysis process, mostly pyrolysis liquid and gases are converted into energy instead of char however, approximately 20% mass of total weight belong to biochar especially in slow pyrolysis process conducted in fixed batch pyrolysis reactor (Kumar et al. 2017). Moreover, this quantity increases in catalytic pyrolysis process since almost all of the catalyst material also remained in the biochar. Additionally, biochar includes many valuable intrinsic inorganic elements, such as Ca, Mg, Al, Fe, K etc., which have catalytic effect on pyrolysis and naturally found in biomass itself. Therefore, biochars should be paid attention as well as pyrolysis liquid and gases for new second- or third-generation solutions in biomass management with sustainable and zero waste approach. To achieve this, characteristics and composition of char should be investigated in detail since biochar structure varies based on the pyrolyzed substance and/or the catalyst used in the catalytic pyrolysis as well as the pyrolysis conditions.

Olive pomace (OP) is lignocellulosic biomass produced from olive oil extraction process, and it contains pieces of olive pit, skin, pulp and oil (Ayadi et al. 2021). It has adverse effects on the environment due to its acidic pH value, high organic matter (lignin, hemicellulose and cellulose) content and non-biodegradable water-soluble phenolic substances (Ayadi et al. 2021). Therefore, waste management of OP and recovery of valuable products from OP with upcycling techniques is important. Thermochemical conversion methods, especially pyrolysis, are among the methods that have been widely used in waste management and recovery of valuable products from OP (Kabakci and Aydemir 2014; Ghouma et al. 2017; Dinc and Yel 2018).

Catalytic pyrolysis has impressive effects on both yield and the quality of thermal decomposition products, since the catalysts cause changes in the reactions occurring during the pyrolysis process (Kumagai et al. 2015; Kim et al. 2002). Catalytic decomposition reactions occur either by protonation of carbon atoms in the polymer chain by protons from Brönsted acid sites, or by removal of hydride ions from the polymer chain by Lewis acid sites (Norea et al. 2012) The resulting ion is stabilized by β-scission, isomerization, or hydrogen transfer reactions. Moreover, catalytic pyrolysis has advantages such as lower energy consumption than non-catalytic, shorter reaction time, and selectivity to high value-added products (Serrano et al. 2000; López et al. 2011; Ali and Qureshi 2013). The catalyst has basically two roles in pyrolysis. First, it lowers the temperature of the pyrolysis process and allows unstable hydrocarbons to combine to form increased amounts of oil. Second, the catalysts mostly create a “cracking” effect that deoxygenates the pyrolysis products by accelerating the production of carbon dioxide and hydrogen. In this way, catalytic pyrolysis contributes to the production of more hydrocarbon-like oil with lower tar and viscous content (Koutinas and Papanikolaou 2011). The functions, disadvantages and/or products of the catalysts vary according to both the structure of the catalyst and the pyrolyzed substance. A wide variety of catalysts, such as activated alumina catalysts, zeolite catalysts, mesoporous catalysts, fluid catalytic cracking catalysts, transition metal catalysts and carbonate-derived catalysts, have been tested in the catalytic pyrolysis process in the literature. Among these, carbonate-derived catalysts are widely used because they are readily available and low cost. Moreover, due to these properties, expensive regeneration processes are not required for them (Koutinas and Papanikolaou 2011). Commonly used carbonate-derived catalysts in pyrolysis studies are dolomite (CaMg(CO_3_)_2_), potassium carbonate (K_2_CO_3_), sodium carbonate (Na_2_CO_3_), iron carbonate (FeCO_3_), calcium carbonate (CaCO_3_), magnesium carbonate (MgCO_3_), calcium oxide (CaO), calcium hydroxide (Ca(OH)_2_), magnesium oxide (MgO) and lithium carbonate (Li_2_CO_3_) (Çaǧlar and Demirbaş 2002; Seçer et al. 2019). Dolomite catalysts show great thermal and mechanical strength and can be used several times with little performance degradation. In one of the studies, the thermal value of the pyrolysis biochar, the amounts of pyrolysis products, the fixed carbon and ash ratios of the pyrolysis biochar, and the stability of the dolomite in the catalytic pyrolysis of OP with dolomite were evaluated and it was investigated that dolomite is quite stable in the pyrolysis process and its catalytic efficiency remains almost constant (Encinar et al. 2009). In the pyrolysis of OP, the effects of K_2_CO_3_ and Na_2_CO_3_ catalysts on the pyrolysis products were observed as irregular, but it was stated that K_2_CO_3_ had a better catalytic effect than the Na_2_CO_3_. Moreover, it has been emphasized that these two alkaline catalysts weaken the C–C bonds with the oxygen transfer mechanism and decrease the activation energy of complex pyrolysis reactions because the alkalis in the structure of the catalysts prevent the formation of stable chemical structures (Çaǧlar and Demirbaş 2002). It has been found that FeCO_3_ increases the gasification efficiency by decreasing the activation energy in both pyrolysis and gasification reactions (Seçer et al. 2019). The effect of Ca(OH)_2_ and CaO on pyrolysis varies depending on both the pyrolysis temperature and the material being pyrolyzed. For example, in the pyrolysis of OP with Ca(OH)_2_, the liquid fraction yield decreased, while the biochar fraction yield increased (Dinc 2018).

Turkey has 5.2 billion m^3^ (13.9 billion tons) of marble reserves and this value represents approximately 33–40% of the world’s marble reserves. Moreover, there are 4 billion m^3^ of exploitable marble, 2.8 billion m^3^ of exploitable travertine and 1 billion m^3^ of granite (Kocabağ 2018). Marble processing wastewater is formed as a result of the use of water to prevent dust and friction heat during marble cutting process. These wastewaters are mostly being treated with physicochemical treatment processes that generates marble sludge. These sludges are either accumulated in the areas belonging to the facility or are tried to be disposed of out of control. However, when the marble sludge is not managed properly, it can cause many types of environmental pollution such as decrement of the soil porosity, air pollution due to the dust in the sludge, reducing groundwater regeneration, etc. (Kocabağ 2018; Ramos et al. 2021). On the other hand, these sludges includes different inorganic compounds, such as CaCO_3_, MgCO_3_, oxides (Al_2_O_3_, NaO, MgO, etc.) and hydroxides (such as Al(OH)_3_) coming from both the marble rock structure and the physicochemical treatment. All these inorganic compounds have catalytic effect on pyrolysis processes as indicated above, therefore significant contribution of marble sludge on the improvement of pyrolysis process is expected in terms of both as catalysing and as generating valuable component recovery. Moreover, when these sludges were launched as a second-generation raw material, the deposition and related environmental problems will also reduce. The studies in the literature mainly focused on the catalytic performance of marble dust in thermochemical processes. Irfan et al. (2021) indicated that gas yield and carbon conversion ratio in the gasification process of municipal solid waste enhanced with Ni-doped marble dust as catalyst. Similarly, H_2_ and CO content in the gas obtained with municipal solid waste gasification process successfully increased with marble dust as catalysts (Amin et al. 2024). Rijo et al. (2022) emphasized that marble dust catalysts in pyrolysis process of forest waste improved the quality of pyrolysis oil by decreasing oxygenated compounds. The literature studies mainly involve marble dust, which is collected from the site as solid particles. However, marble sludge used as catalyst in presented study differed from the literature by consisting of not only marble dust but also its suspension in cooling water together with other processing wastes, oxides/hydroxides structures forming in physicochemical treatment process of marble wastewater and alkaline-earth elements besides calcite-dolomite. Furthermore, there are not any study that present the potential usage of OP catalytic biochars obtained with marble sludge catalysts produced in this study in different areas with new and environmental approach by evaluating characteristic qualities.

In this regard, in this study, marble sludge obtained from marble processing wastewater physicochemical treatment process was added at different dosages and pyrolysis temperature in OP pyrolysis. The effects of these sludges on the potential green applications of OP pyrolysis biochar and pyrolysis product yields were investigated in detail. Moreover, the utilization of OP catalytic biochar in new usage areas and different purposes were reported based on characteristic qualities of biochars. By virtue of this study, it was revealed that marble sludge catalyst can be utilized as low-cost, sustainable catalyst successfully in OP pyrolysis by gaining favor both waste management and circular economy.

## Materials and methods

### Raw OP and marble sludge catalyst

The olive pomace (OP) samples studied in the study were obtained from the Ernar Inc. Co. Ltd olive oil production facility operating in Mersin/Erdemli. Olive squeezing is carried out in 2-phase at the facility, so the amount of OP produced in the facility is higher than olive mill wastewater (OMWW). Approximately 3000 tons of olives are pressed in one season at the facility and 2100–2500 tons/season pomace is obtained. OP sample used in this study had sticky and slime structure which includes olive pits particles with a size of maximum 1–1.5 cm.

**Fig. 1:**
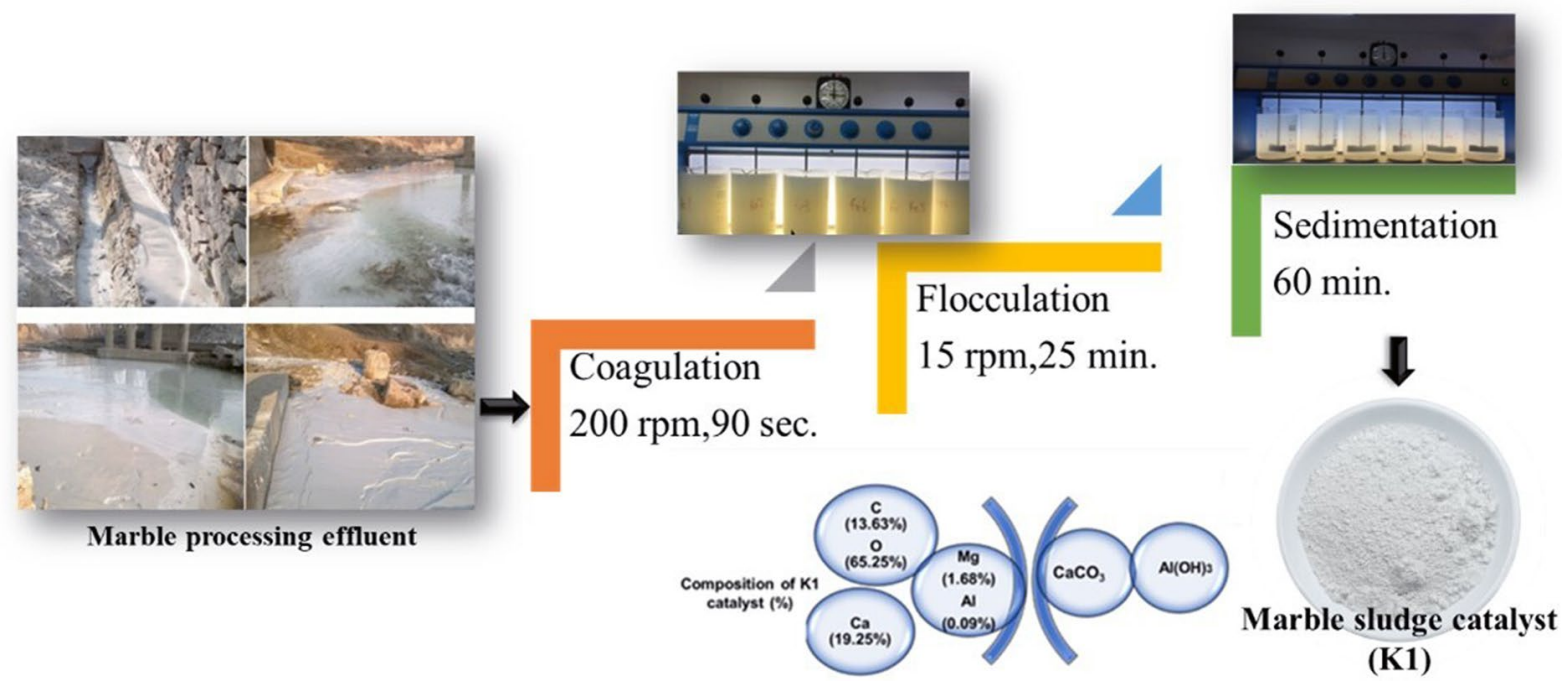
Preparation of K1

Marble processing effluent samples were taken from the marble processing plant of REMAR Inc. Co., operating in Konya (Turkey). Marble sludge catalyst used in the pyrolysis was generated by applying physicochemical treatment process to the marble processing effluent with the alum chemical. Preparation of marble sludge catalyst (K1) was presented in Fig. 1. Optimum conditions for physicochemical process (coagulation-flocculation and sedimentation) were determined in previous study of the authors (Onen et al. 2023). K1 mainly comprises of CaCO_3_ and Al(OH)_3_ but it includes also inorganic elements coming from origin of the marble stone (Fig. 1). Composition of K1 catalyst was taken from the previous study of the authors (Onen et al. 2023; Goktepeli and Yel 2023).

### Pyrolysis experiments and analyses

**Fig. 2:**
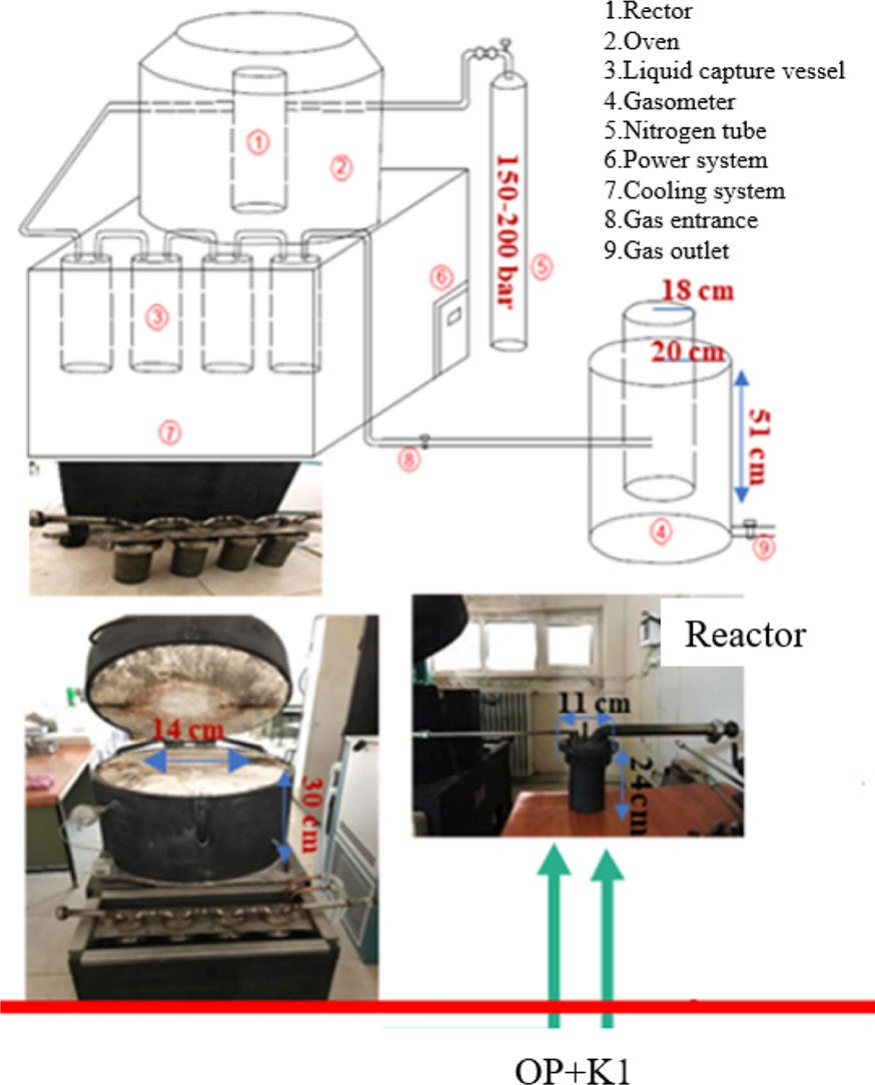
Lab-scale fixed bed batch pyrolysis system

OP samples were pyrolyzed in the lab-scale fixed bed batch pyrolysis system, which has power control, heating, cooling-capture and gas collection parts (Fig. 2), with and without K1 catalyst. In catalytic pyrolysis experiments, four catalyst dosages (10%, 20%, 30% and 50%) and three final pyrolysis temperatures (300 °C, 500 °C and 700 °C) were studied with at least two replicates and at 5 °C min^−1^ heating rate. The system was operated as pyrolysis with no further retention at the target temperature. Pyrolysis experiment durations were 60, 100 and 140 min for 300 °C, 500 °C and 700 °C, respectively. K1 catalyst and OP wastes were mixed homogeneously in specified proportions as total weight was 100 g and OP-K1 mixtures were placed into the pyrolysis reactor which was made of stainless steel and durable up to 900 °C (Fig. 2). NiCr–Ni thermocouple was used for controlling the internal temperature of the pyrolysis system and N_2_ was used as carrying gas to satisfy oxygen-free environment into the system. At the end of the pyrolysis process (i.e., reaching the target pyrolysis temperature), pyrolysis biochar, liquid and gas products were collected. Pyrolysis biochars and liquids were weighed as a result of the experiment. The collected pyrolysis gas quantities, on the other hand, were recorded as the gas volume collected in the gasometer. Pyrolysis product yields were presented as bar graph. Moreover, Two-Way ANOVA test of IBM-SPSS Statistic program (significance level α = 0.05) was used to evaluate the effects of K1 and temperature on pyrolysis product yields.

Thermogravimetric (TGA), Fourier transform infrared spectroscopy (FTIR), elemental, XRD, SEM, BET, and biochar surface acidity, moisture and ash content, and calorific value analyzes were performed for the characterization of the obtained pyrolysis biochars. PerkinElmer—TGA4000 Model device was used for TGA analyses. During the analysis, nitrogen gas was continuously supplied to the device at a rate of 20 mL min^−1^ and the target TGA temperature was set to 900 °C and the heating rate to 20 °C min^−1^. FTIR analyzes were performed using the potassium bromide (KBr) method on a Thermo Scientific Nicolet iS5 FT-IR spectrometer. The sample was added in the range of 0.2–1% and mixed with KBr in such a way as to obtain a fully homogeneous mixture. Pellet was obtained from KBr-sample mixture with the help of metal disc and analysis was performed with the help of ID 1 Transmission Module in the FTIR device. LECO/TruSpec Micro device was used for elemental analysis of samples. C, H, N and S fractions were determined with the combustion of samples at 950–1300 °C temperature in the pure oxygen environment. As a result of combustion, carbon, CO_2_, H_2_O, N_2_ and sulfur were transformed into SO_2_ gases and the device determines the percentage of C, H, N, S in the sample based on these gases. XRD patterns were obtained by using Rigaku Brand SmartLab Model XRD device with D/teX Ultra 250 detectors, giving information about the phases of the samples, the amounts of the phases and the crystal sizes. As for XRD analysis, 40 kV, 30 mA X-ray was used at 10.0000–60.0000° scan range with 3.0352° min^−1^ scan velocity. BET analyzes were performed with Quantachrome-Autosorb iQ Station 2 model device, 9 mm w/o rod cell type and nitrogen as analysis gas. In the BET analysis, the exit gas time was set to 8 h, and the degassing temperature was set to 300 °C with 6.58e^−05^ 1/mmhg pressure. SEM images were examined in JEOL-JSM-6610 model device, at 15 kV acceleration voltage, SEI signal, standard filament current and probe current conditions. The determination of total solids (TS) and volatile solids (VS) in OP was made according to the SM 2540 B and SM 2540 E method, respectively (APHA, AWWA, WEF 2012). Inorganic substances in OP were determined by using the Thermo Fisher Scientific 6000 series ICP-OES instrument after microwave pretreatment. EPA 3051A method was used for pretreatment in microwave by mixing 9 mL of HNO_3_, 3 mL of HCl with 0.5 g of OP and setting microwave combustion system at 180 °C and 30 min, respectively (U.S. EPA 2007). After pretreatment, mixture was analyzed with the ICP-OES device with argon flow rate of 0.5 L min^−1^ and at 6 bar pressure (EPA 1994). Moisture and ash content was determined according to ASTM standards D3173-85 and D3174-82. Heating values of coke samples were measured by using O_2_ gas at 15–20 atm pressure in Leco Brand AC-350 Model Calorimeter Device.

The quantity of the potassium hydroxide needed for neutralization of the free acid in the sample represent the acid value. In order to measure biochars’ acid value, isopropyl alcohol-toluene mixture prepared at a ratio of 1:1 by volume was added to the sample as solvent and 1% phenolphthalein solution prepared with isopropyl alcohol was used as indicator. Then, the sample was titrated with 0.1% N KOH solution until a pink color was obtained. The acid value was calculated with the Eq. (1) (Boz and Kara 2009; Kaşikçi 2009)

1 $${\text{Acid \,Value}}\left( {{\text{mmol}}/{\text{g}}} \right) = \left( {{\text{A}} - {\text{B}}} \right){\text{N}}/{\text{W}}$$

where A: volume of standard alkali used in titration (mL), B: volume of standard alkali used in titration of blank (mL), N: normality of standard alkali, W: amount of sample (g).

## Results and discussion

### Olive pomace characteristics

**Fig. 3:**
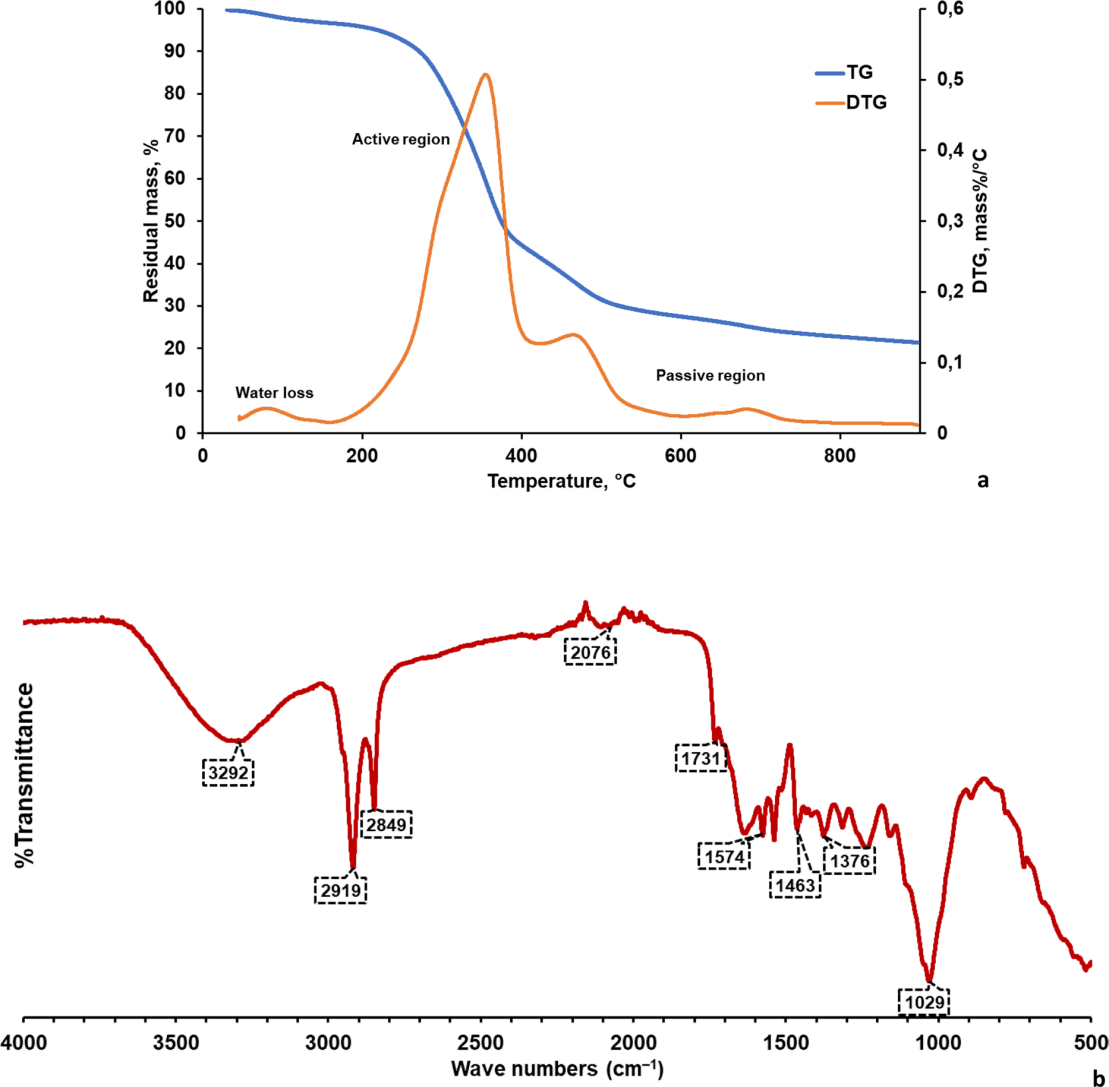
**a** TG/DTG graph, and **b** FTIR spectrum of the dried and grinded OP sample

Characteristics of K1 basically resemble the calcite and dolomite structures and it includes mainly Ca (19.25%), C (13.63%), and O (65.25%) elements in the structure (Onen et al. 2023). TG and FTIR analysis were conducted for OP characterization, and TG and FTIR graphs were given in Fig. 3. While the mass loss of pomace initially showed a low decrease at temperatures approximately up to 100 °C, it increased at high temperatures and lost about 70% of its mass at 600 °C (Fig. 3a). This result was consistent with the literature (Ghouma et al. 2017; Bennini et al. 2019). Increment in the mass losses with temperature can be attributed to chemical reactions that lead to the release of volatile substances and tar. The DTG plot of pomace (Fig. 3a) can be evaluated in three different regions as water loss, active and passive regions. The first region where mass loss occurs is the drying phase of the pomace and the loss of water in the pomace started from room temperature and continued until about 177 °C (Fig. 3a). The active region in the pomace thermogram is located between 177 and 380 °C. This region is one of the most important parts of the pyrolysis process and most of the volatile substances originating from the degradation of OP are formed in this region. This degradation in the active site is due to hemicellulose and cellulose in OP structure. While hemicellulose starts to degrade at lower temperatures (157–357 °C) with the release of volatile substances with light molecular weight, cellulose degrades in the range of 240–390 °C (Khalideh et al. 2017; Ghouma et al. 2017). Therefore, it can be stated that the decomposition in the active region in Fig. 3a was caused by hemicellulose and cellulose in the structure of pomace. As for DTG plot, overlapping in the DTG peaks was observed and the peaks could not be defined separately since the temperature range at which the most degradation of OP (250–350 °C) covers both hemicellulose and cellulose degradation temperature (Khalideh et al. 2017). Contrary to hemicellulose and cellulose, lignin was more difficult to decompose because it has a rich chemical structure with several branched aromatic rings. Therefore, its degradation covers a much wider temperature range in both active and passive regions in the range of 160–600 °C and heavier volatile components are formed (Yang et al. 2007). Therefore, lignin degradation was mostly observed in the passive region in the pomace thermogram. The peaks observed in the passive region of Fig. 3a can be attributed to the degradation of different organic compounds in OP structure having high molecular weight such as Guaiacol type (G-type), Syringol type (S-type) and Phenol type (H-type) phenolic compounds (Dinc 2018).

OP contains water, lipids (residual oil), polyunsaturated fatty acids, phenols, and some inorganic compounds. Cellulose, hemicellulose, and lignin are the main components. In addition to these, fats and proteins exist in trace quantities (Wedyan et al. 2017; Ying et al. 2017). In Fig. 3b, FTIR spectrum peak mainly changed in the range of 3299–2849 cm^−1^ and 1731–517 cm^−1^ wavenumbers. In the spectrum, 2919 and 2849 cm^−1^ wavenumbers represent C–H stretch of alkane aliphatic methylene and methyl groups present in the lignin, while O–H stretch of water, cellulose, hemicellulose, and phenolic-aliphatic hydroxy compounds of lignin was observed at 3299 cm^−1^. C=O stretching of triglycerides and alkyl ester (pectin) appeared at 1731 cm^−1^. COO− asymmetric stretching (pectin ester group), C=O stretching of protein primary and secondary amides, N–H bending of amine, or aromatic ring C=C stretching at 1633 cm^−1^, N–H bending vibration coupled to C–N stretching in protein at 1576 cm^−1^, amino group at 1538 cm^−1^, O–H deformation or C–O stretching of phenolic compounds at 1403 cm^−1^, C–O stretching of aromatic ester at 1314 cm^−1^, aryl–O stretch of aromatic ethers and C–O stretching of hemicellulose or syringyl ring in lignin at 1238 cm^−1^ were detected (Ying et al. 2017; Bekiaris et al. 2020). Calero et al. (2013) reported that esters in the lignin structure are between 1520 and 1540 cm^−1^ wavenumbers. The peaks between 1160 and 1000 cm^−1^ may originate from C–O–C and O–H vibrations in polysaccharides or C–N bond (Rizzi et al. 2017) (Fig. 3b). All these indicated that OP is a complex chemical structure as the combination of number of aliphatic, aromatic, unsaturated and complex organics. This property makes it available to number of chemical reactions to produce diverse compounds upon degradation.

Inorganic content of OP sample used in this study was given in Table 1. Since OP is the pulp obtained as a result of the squeezing of olives, it contains water as well as oil. However, the moisture content of OP varies according to the pressing method. Since the olive oil production factory of Ernar Inc. Co. Ltd (Mersin-Erdemli), from where OP was supplied, is applying 2-phase olive squeezing, the moisture content of OP is high (Table 1). The moisture levels of OPs released in 2-phase olive squeezing facilities in Turkey are in the range of 60–75% (TÜBİTAK 2015). The pH value of OP is moderately acidic due to the components (such as phenols, and fatty acids) in its structure (Gianico et al. 2013).

**Table 1:** Characteristics of OP

Proximate analysis	This study	Other studies (Morillo et al. 2009; TÜBİTAK 2015; Al-Addous et al. 2017; Dinc 2018; Bennini et al. 2019)
Heat value (kcal kg^−1^)	3500–4000	4000–5000
Moisture (%)	65.0	9.6–71.4
Ash (%)	4.2	–
pH	5.1	4.9–6.8
TS (%)	54.0	57–90
VS (%)	89.0	55–88
Ultimate analysis		
C (%)	26.7	25–55
H (%)	3.7	6–8
N (%)	1.5	0.7–2
S (%)	–	< 0.1
Inorganic content		
K (mg kg^−1^)	17,137.5	6300.0–29000.0
Ca (mg kg^−1^)	7250.0	2300.0–12000.0
P (mg kg^−1^)	1561.0	300.0–1500.0
Mg (mg kg^−1^)	1656.5	500.0–1700.0
Fe (mg kg^−1^)	469.9	526.0–2600.0
Na (mg kg^−1^)	580.0	200.0–1000.0
Al (mg kg^−1^)	311.1	18.9–273.5
Sn (mg kg^−1^)	155.3	**–**
Cu (mg kg^−1^)	31.2	**–**
Zn (mg kg^−1^)	22.1	10.0–27.0
Mn (mg kg^−1^)	20.5	13.0–67.0
Ti (mg kg^−1^)	15.8	**–**
Se (mg kg^−1^)	13.8	**–**
Ba (mg kg^−1^)	5.4	0.28–5.7
Ce (mg kg^−1^)	2.6	–
Cr (mg kg^−1^)	2.1	0.5–2.26
Ni (mg kg^−1^)	2.7	0.7–3.3
B (mg kg^−1^)	1.7	**–**
Mo (mg kg^−1^)	0.8	0.1–1.8
Pb (mg kg^−1^)	0.6	**–**
Ag (mg kg^−1^)	0.6	**–**
Li (mg kg^−1^)	0.5	**–**
Hg (mg kg^−1^)	0.3	**–**
Co (mg kg^−1^)	0.2	**–**
Cd (mg kg^−1^)	0.05	**–**
Sb (mg kg^−1^)	0.01	**–**
∑Inorganic (mg kg^−1^)	29,242.3	**–**

The solids ratio of OP is quite high (Table 1). While the percentage of total solids (TS) in OP is 54, the percentage of volatile solids (VS) is 89. A wide range for the solids content of OP is available in the literature. The percentages of solids in the OP vary depending on the rainfall and average temperatures of the countries and/or regions where olive oil is produced (Bennini et al. 2019). The VS value in the OP biomass used in the study is quite high compared to TS and this is comparable with the VS value of other lignocellulosic agricultural wastes in the literature (between 70 and 80%) (Bennini et al. 2019). The ash value of OP is quite low compared to TS and VS. The high C and O elemental ratios constitute approximately 90% of the OP structure (Bennini et al. 2019). This explains the low percentage of ash in OP. As a lignocellulosic biomass, OP is very rich in terms of inorganic elements. The macro inorganic elements in OP are K, Ca, Mg, P, Na, Fe, Al, Sn; while micro inorganic elements are Cu, Zn, Mn, Ti, Se, Ba, Ce, Cr, Ni, B, Mo, Pb, Ag, Li, Hg, Co, Cd and Sb (Table 1). K, P, Ca and Mg are the major inorganic elements that must be found in the soil for the olive fruit to grow in a healthy way. In the deficiency of these inorganic elements, it is observed that the leaves of the olive plant fall off, turn yellow, decrease in fruit size, and stop the growth of the shoots (Azimi et al. 2015). Therefore, these inorganic elements are sufficient in the soils of the regions where olive trees grow, and for this reason, K, P, Ca and Mg are among the macro inorganic elements in the OP biomass.

### Effects of marble sludge catalyst (K1) on the thermal stability of non-pyrolyzed OP

**Fig. 4:**
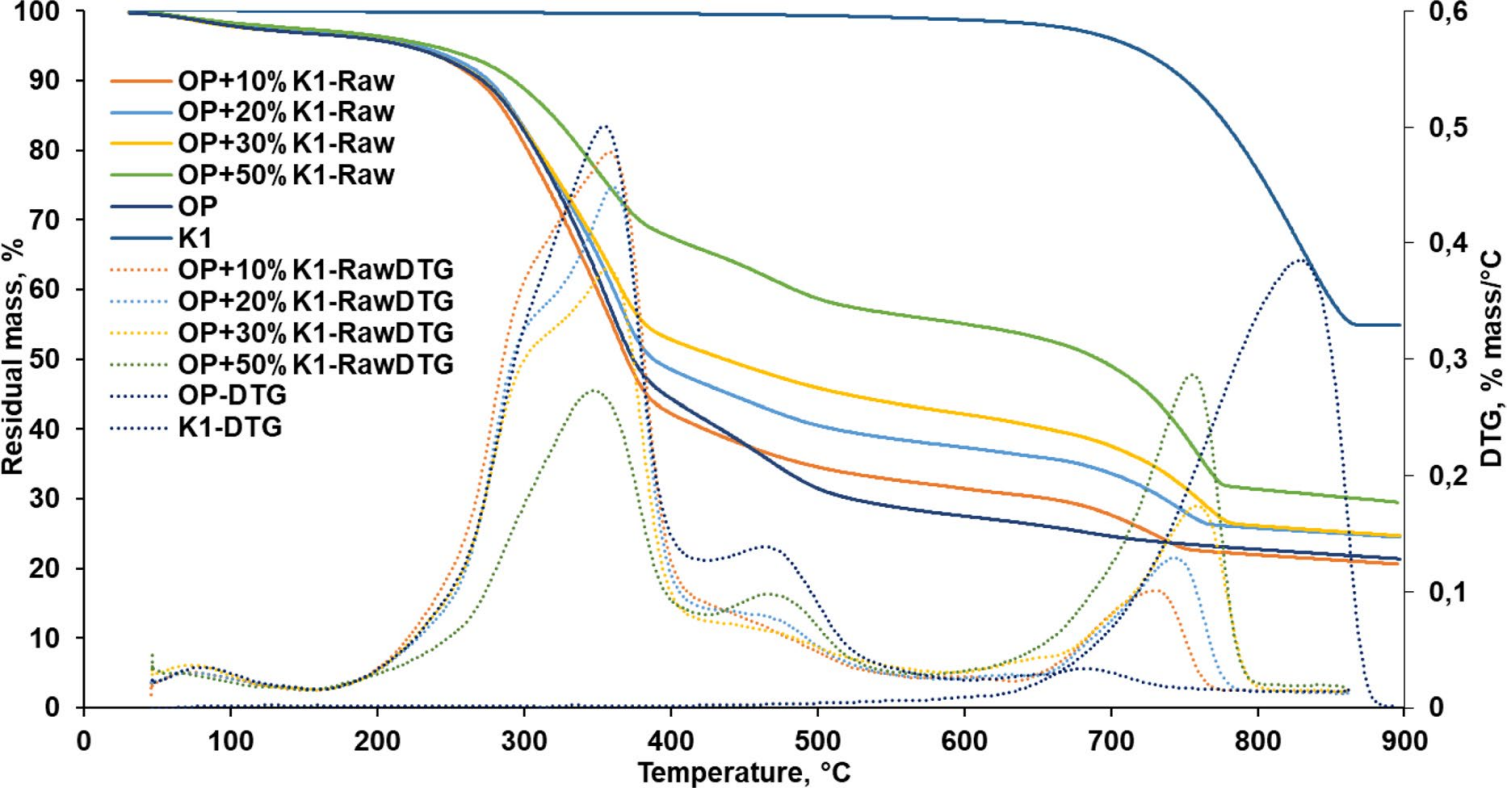
TGA thermograms of OP, K1 and OP+K1 mixture

OP was mixed with K1 in varying weight ratios and pyrolyzed at different temperatures. The thermogravimetric behavior of these OP+K1 mixtures before pyrolysis was investigated, as it will help to interpret the possible catalytic effect of K1 in the pyrolysis process (Fig. 4). The thermal resistance values determined according to the TGA data in Fig. 4 were given in Table 2. OP has three thermal degradation steps: volatilization of moisture and low molecular weight volatile substances, hemicellulose and cellulose degradation, lignin degradation and formation of residue (Figs. 3, 4). In TGA thermograms of OP+K1 mixtures, a fourth step, which is the decomposition of CaCO_3_ and pyrolysis biochar formation stage, was observed differently from OP thermogram. Especially at high K1 dose (50%), the fourth stage caused by the breakdown of CaCO_3_ was more clearly observed (Fig. 4). The thermal resistance of K1 was quite high compared to OP and the residual value at 900 °C was about 55% (Table 2). Therefore, the increase in the K1 dose in the OP+K1 mixture also increased thermal resistance. The highest residue amount in TGA analysis was observed at 50% OP + 50% K1 mixture. This can be connected to the absorption of CO_2_ by CaO which forms during degradation of CaCO_3_ in K1 structure. In TG analysis, there is an inert environment and various gases, including CO_2_, are produced during the thermal decomposition of biomass [Eq. (2)]. The structure of K1 is similar to dolomite (CaMg(CO_3_)_2_) and CaO is released as a result of the decomposition of dolomite in two stages in an inert medium [Eqs. (3) and (4)]. Therefore, the mass reduction in OP+K1 mixtures is delayed compared to OP alone and the amount of residue increases (Sun et al. 2017) as a result of absorption of CO_2_ formed during the biomass decomposition by CaO.

2 $${\text{Pyrolysis}}:{\text{ Biomass}} \to {\text{Gases}}({\text{H}}_{{2}} + {\text{CO}} + {\mathbf{CO}}_{{\mathbf{2}}} + {\text{H}}_{{2}} {\text{O}} + {\text{CH}}_{{4}} + {\text{C}}_{{\text{n}}} {\text{H}}_{{\text{m}}} ) + {\text{Tar}} + {\text{Biochar}}$$

3 $${\text{CaMg}}\left( {{\text{CO}}_{{3}} } \right)_{{2}} \to {\text{CaCO}}_{{3}} + {\text{MgO}} + {\text{CO}}_{{2}}$$

4 $${\text{CaCO}}_{{3}} \to {\mathbf{CaO}} + {\text{CO}}_{{2}}$$

5 $${\mathbf{CaO}} + {\mathbf{CO}}_{{\mathbf{2}}} + {\text{H}}_{{2}} {\text{O}} \to {\text{Ca}}\left( {{\text{OH}}} \right)_{{2}} + {\text{CO}}_{{2}} \to {\text{CaCO}}_{{3}} + {\text{H}}_{{2}} {\text{O}}$$

**Table 2:** Thermogravimetric findings of OP, K1 and OP+K1 mixtures without pyrolysis

Sample		Thermal resistance values							Residual at 600 °C (%)	Residual at 900 °C (%)
Type	K1 dosage (%)	DT_*1_ (°C)	DT_*2_ (°C)	DT_*3_ (°C)	DT_*4_ (°C)	T_5_ (°C)	T_10_ (°C)	T_50_ (°C)		
OP	**–**	172.2	395.8	486.2	**–**	219.3	271.4	374.1	27.5	21.4
K1	**–**	613.4	**–**	**–**	**–**	713.4	751.0	**–**	98.7	54.9
OP+K1 mixture	10	175.3	395.7	494.2	624.0	223.4	268.4	369.9	31.5	20.6
	20	176.3	394.6	500.0	621.8	227.4	275.7	387.8	37.4	24.5
	30	183.1	392.8	501.8	616.0	219.9	272	436.2	42.1	24.7
	50	195.3	384.6	514.5	614.2	236.1	292.9	691.1	55.1	29.5

DT_*1_ first decomposition temperature; DT_*2_ second decomposition temperature, DT_*3_ third decomposition temperature, DT_*4_ fourth decomposition temperature, *T*_*5, 10, 50*_ temperatures at which 5%, 10% and 50% decomposition occurs

According to Table 2, the first degradation temperatures increased with the increment of the K1 dose in the mixture and rose to 195.3 °C for the highest K1 dose. The first degradation temperatures for the mixtures correspond to the degradation of cellulose and hemicellulose in the OP structure, and the second degradation temperatures correspond to the temperatures at which both lignin and cellulose degradation. It is stated in the literature that ketone, aldehyde, and ether groups in the structure of OP are degraded at temperatures where 10% decomposition occurs (Tac 2020). Lignin degrades over a wider temperature range than hemicellulose and cellulose. Although the degradation temperatures of lignin vary according to the Syringol type phenol (S)/Guaiacol type phenol (G) ratio in its structure, it generally starts to decompose in the range of 200–400 °C with primary pyrolysis reactions, and the maximum degradation is mostly observed around 350 °C (Kawamoto 2017). At temperatures higher than 400 °C, it continues to decompose by secondary pyrolysis reactions. According to Table 2, the secondary decomposition temperatures decreased as the K1 dose increased in the mixture. This indicates that K1 has a catalytic effect on the thermal decomposition of OP and it decreases decomposition temperature of lignin + cellulose compared to OP alone. In addition to these, a significant increase was observed in T_5_, T_10_, and T_50_ values by increasing the ratio of K1 in the mixture from 10 to 50% due to the high thermal resistance of K1 (Table 2).

### Pyrolysis product fractions

**Fig. 5:**
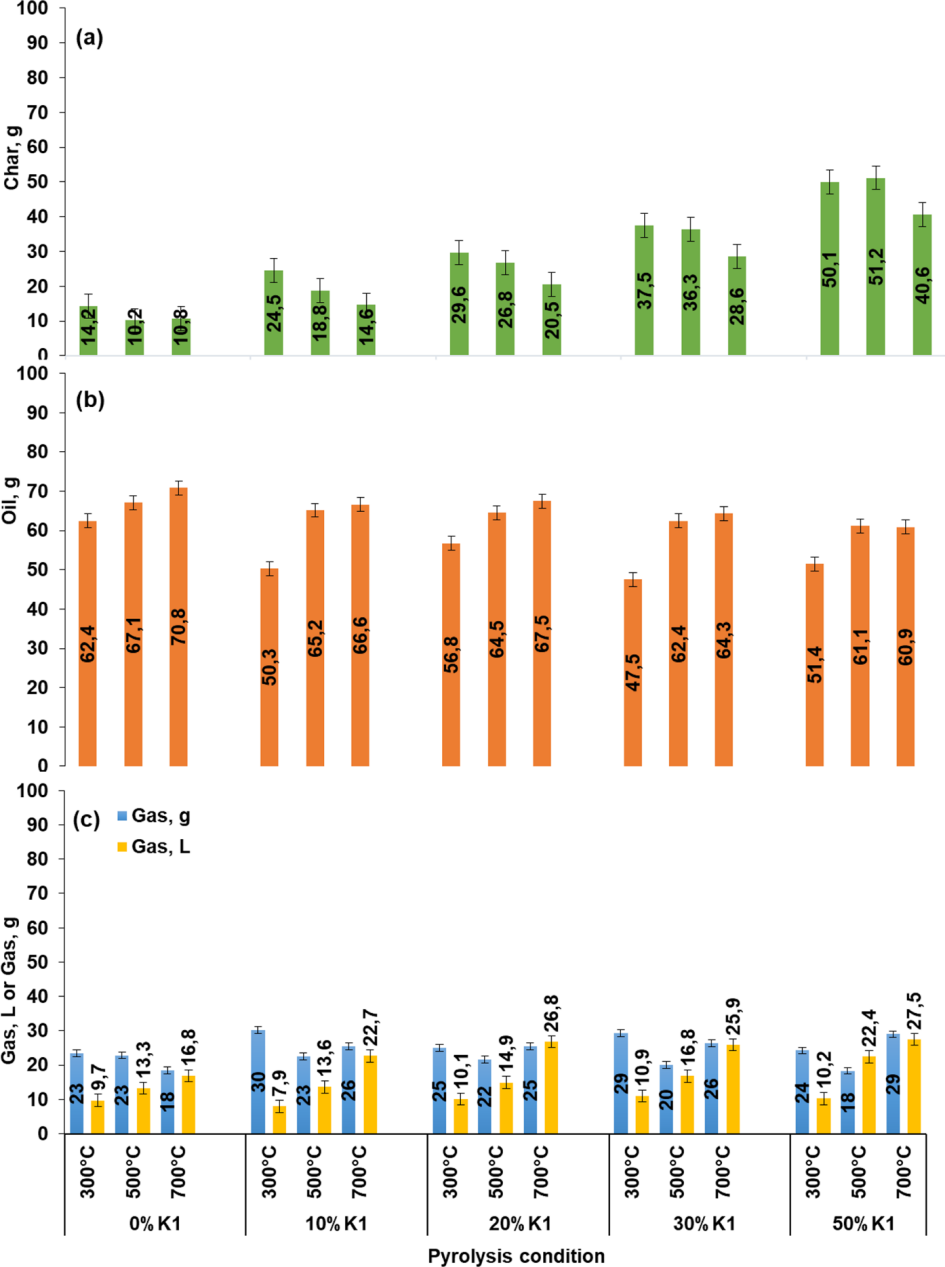
Pyrolysis product yields of OP+K1 mixture: **a** Total solid product including the added K1, **b** Pyrolysis liquid, **c** Pyrolysis gas excluding carrying gas volume

Pyrolysis product yields of the mixtures of OP with K1 in different ratios at 300, 500, 700 °C and 5 °C min^−1^ heating rate were given in Fig. 5. The lowest amount of pyrolysis biochar was observed in the pyrolysis of OP alone for all the temperature values selected within the scope of the study. For the mixtures of OP and K1, the amount of biochar increased as the K1 dose increased for the same temperature (Fig. 5). This can be attributed that the structure of the K1 catalyst is similar to dolomite (Ca·Mg(CO_3_)_2_) and the decomposition of dolomite increases at higher temperatures than the studied pyrolysis degrees (700 °C and above) (Olszak-Humienik and Jablonski 2015). For the same K1 dose, the increase in temperature mostly decreased the amount of pyrolysis biochar and the lowest biochar amounts were obtained at 700 °C (Fig. 5). For this reason, it can be said that for both catalytic and non-catalytic OP pyrolysis, the increase in temperature decreases the amount of the pyrolysis biochar due to increased decomposition. This decrease in pyrolysis biochars with temperature increase for the same K1 doses is also similar to the OP pyrolysis studies with dolomite catalyst in the literature (Encinar et al. 2009; Taralas and Kontominas 2006). The use of K1 catalyst in pyrolysis caused a slight decrease in the liquid product yield. As for pyrolysis liquid, increment in the K1 ratio from 20 to 50% in the mixture resulted in decrement of pyrolysis liquid quantity for all studied temperatures. However, K1 doses at 10% and 20% did not make a significant change in the liquid quantity (Fig. 5). Similarly, in a study in which catalytic pyrolysis of pomace with dolomite was carried out, it was studied in the temperature range of 500–700 °C and they observed that increasing the catalyst dose at these temperatures decreased the amount of pyrolysis liquid (Encinar et al. 2009).

It was observed that the increment of temperature for the same catalyst doses mostly caused an increase in the amount of pyrolysis liquid, except for 300 °C (Fig. 5). This can be explained with the uncomplete pyrolysis reactions in 300 °C temperature. These results of the study consistent with the previous study of the authors which was emphasized that the amount of pyrolysis liquid obtained at 450–600 °C pyrolysis temperature and 5 °C min^−1^ heating rate of pomace increased with the increment of the temperature (Dinc and Yel 2018). The gas volumes obtained in the pyrolysis of mixtures mostly increased regularly as the pyrolysis temperature increased. However, in pyrolysis with 0% catalyst, there was a more irregular change in the quantity of gas at 300 °C (Fig. 5). This can be explained by the fact that 300 °C coincides with the decomposition temperature ranges of hemicellulose and cellulose in the pomace structure. The increase in pyrolysis gases at higher temperatures can also be attributed to more gas formation with the increase of thermal cracking reactions. These findings are consistent with the pomace studies in the literature (Taralas and Kontominas 2006; Encinar et al. 2009). The pyrolysis gases mostly increased with the increase of the K1 dose in the mixture at the same pyrolysis temperatures; but an exact trend of change was not observed in some K1 dosage. This can be attributed that the degradation reactions of the K1 catalyst depend on more variable factors in the pyrolysis of pomace. Moreover, since the pomace pyrolysis was carried out in a fixed bed batch pyrolysis system, the CO_2_ formed in the reactor during the pyrolysis may affect the process.

The effects of K1 and temperature on pyrolysis product yields were also tested by using Two-Way ANOVA test of IBM-SPSS Statistic software (significance level α = 0.05). According to the F-test of pyrolysis product yields (Table 3), significance value for all pyrolysis product was higher than 0.05, which indicates that the variances are evenly distributed. When the importance of temperature and catalysts factor on the pyrolysis product yields were evaluated (Table 4), it can be stated that both factors have a significant effect since significance value for K1 and temperature was lower than 0.05 for pyrolysis char, liquid, and gas. Therefore, both bar graph and statistical analysis of pyrolysis product yields showed that both catalyst and pyrolysis temperature have an important effect on the product yields.

**Table 3:** F-test for OP-K1 mixture pyrolysis product yields

Products	F test for heteroskedasticity^a,b,c^			
	F	df1	df2	Sig.
Pyrolysis char	0,529	1	43	0,471
Pyrolysis liquid	0,510	1	43	0,479
Pyrolysis gas	3,737	1	43	0,060

^a^Dependent variable: char, pyrolysis liquid and gas

^b^Tests the null hypothesis that the variance of the errors does not depend on the values of the independent variables

^c^Predicted values from design: Intercept + Temperature + K1 + Temperature*K1

**Table 4:** Tests of between-subjects effects

Source	Type III sum of squares	df	Mean square	F			Sig.			Partial eta squared
Dependent variable: char										
Corrected model	7472,473^a^	14	533,748	6411,132			< 0,001			1,000
Intercept	34405,172	1	34405,172	413258,787			< 0,001			1,000
Temperature	522,142	2	261,071	3135,865			< 0,001			0,995
K1	6822,618	4	1705,654	20487,521			< 0,001			1,000
Temperature* K1	127,713	8	15,964	191,754			< 0,001			0,981
Error	2,498	30	0,083							
Total	41880,143	45								
Corrected total	7474,971	44								
Dependent variable: liquid										
Corrected model	1873,199^b^	14	133,800	7,111			< 0,001			0,768
Intercept	165236,015	1	165236,015	8781,887			< 0,001			0,997
Temperature	1167,288	2	583,644	31,019			< 0,001			0,674
K1	255,308	4	63,827	3,392			0,021			0,311
Temperature*K1	450,603	8	56,325	2,994			0,014			0,444
Error	564,466	30	18,816							
Total	167673,681	45								
Corrected total	2437,665	44								
Dependent variable: gas										
Corrected model	1962,978^c^	14	140,213	862,068			< 0,001			0,998
Intercept	12581,728	1	12581,728	77356,031			< 0,001			1,000
Temperature	1537,540	2	768,770	4726,616			< 0,001			0,997
K1	257,498	4	64,374	395,793			< 0,001			0,981
Temperature*K1	167,940	8	20,993	129,068			< 0,001			0,972
Error	4,879	30	0,163							
Total	14549,586	45								
Corrected total	1967,858	44								

^a^R squared = 1,000 (adjusted R squared = 1,000)

^b^R squared = 0,768 (adjusted R squared = 0,660)

^c^R squared = 0,998 (adjusted R squared = 0,996)

### Potential of catalytic biochars via their surface morphologies

#### Biochar morphology

**Fig. 6:**
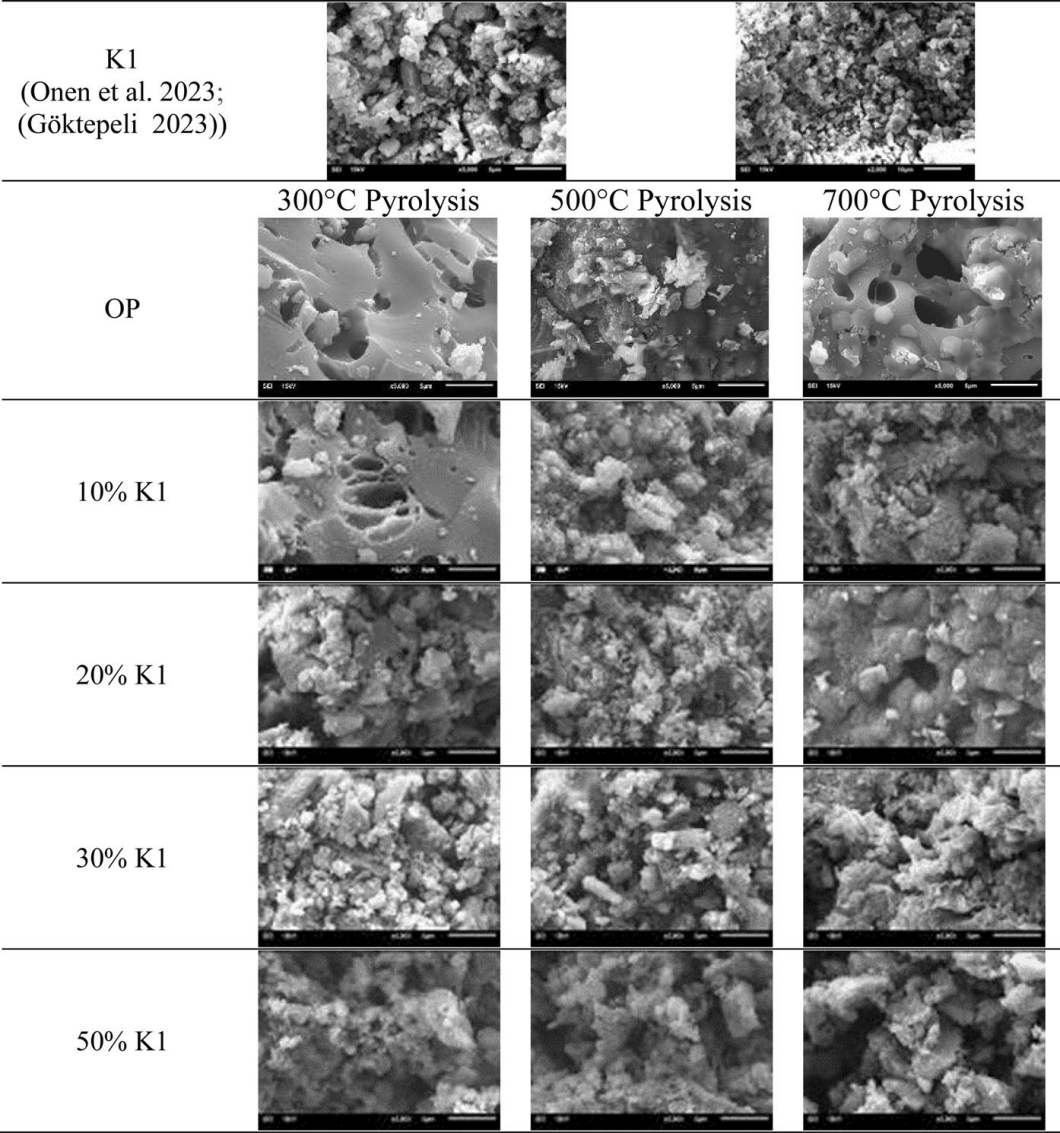
SEM images of K1 and pyrolysis biochars (5 K magnification)

To determine potential uses of the produced catalytic biochars, first, their surface morphologies were examined. SEM images of K1, OP and biochars of OP+K1 mixture were given in Fig. 6. Surfaces of 300 °C pyrolysis biochars up to 30% K1 dose were smooth and resembled an oily adherent mass. Therefore, it can be concluded that OP was not completely decomposed at that temperature with lower catalyst doses. Oily, flat but relatively rough biochar surfaces were observed compared to the biochars obtained without catalyst at 300 °C pyrolysis temperature and with 10% K1. This shows the catalytic effect of K1 on the decomposition of OP. Even with low pyrolysis temperature at 30% and 50% K1 doses, it showed the catalyst effect, and this enhanced the degradation and by enhanced separation of volatile compounds to fluid portion a porous structure was obtained. When the K1 dose was increased to 30%, the temperature increment did not have remarkable effect on biochar surface characteristics. This situation can be attributed to the completion of degradation of OP. Although large agglomerates in the structure of the biochar obtained from pyrolysis did not become smaller at 300 °C, the size of the biochar particles decreased, and their number increased. As for 500 °C and 700 °C pyrolysis biochars, roughness and pores of the biochars increased since more degradation occurred. Some amorphous carbon structures are also formed during pyrolysis due to the decomposition of cellulose (Zhao et al. 2017). It has been reported that amorphous carbon structures can form micropores (Vamvuka and Sfakiotakis 2011; Tomczyk et al. 2020). Higher pyrolysis temperature is needed for the volatile matter releasing and generation of more pores (Shaaban et al. 2014). Moreover, the porosity increased slightly at 500 °C and sintering was observed by aggregation of the particles at 700 °C. As for 50% K1 dose, porosity of the biochars increased as compared to 30% K1 for all studied temperatures. However, temperature increment at this dose did not significantly affect biochar morphology (Fig. 6).

**Fig. 7:**
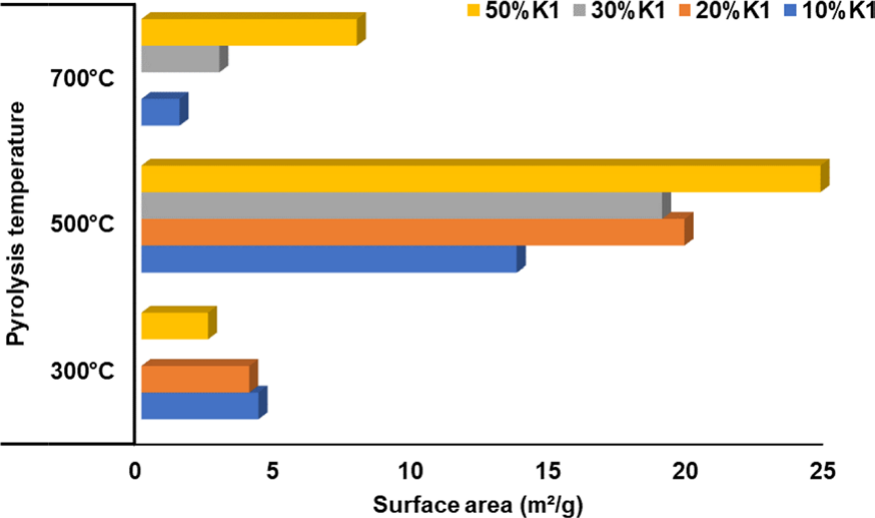
Surface area of biochars obtained at varying K1 dose and pyrolysis temperatures of OP+K1 mixtures

The visual observations from SEM images indicate the differences in surface morphologies, however, a systematic effect of K1 dose or pyrolysis temperature on BET surface areas of biochars cannot be defined (Fig. 7). For 10–30% catalyst doses, the surface area of the biochars obtained at the pyrolysis temperature of 700 °C increased compared to the biochars obtained at 300 °C. A known fact that biochar surface area and porosity are affected from increment of pyrolysis temperature in which is due to degradation of organic substances and micropores generation (Katyal et al. 2003). The surface area of biochar increases with increasing temperature. Pore-blocking substances are removed or thermally broken down with increasing pyrolysis temperature, thereby the externally accessible surface area increases (Rafiq et al. 2016). Pyrolysis can increase surface area and pore volumes through the progressive degradation of organic materials (cellulose, lignin) and the formation of channel structure (Li et al. 2013; Zhao et al. 2017). Furthermore, the destruction of aliphatic alkyl and ester groups and exposure of the aromatic lignin core to higher pyrolysis temperatures can result in increased surface area (Chen and Chen 2009). The lignocellulosic structure within the OP decomposed better at high pyrolysis temperatures, resulting in more porous pyrolysis biochar. Due to the increased porosity in the biochar structure with the catalyst dose, especially at 500 °C pyrolysis temperature, the surface area of the pyrolysis biochars increased (Fig. 7). This is an important result for the usage of biochars as anodes for lithium-ion batteries. Using chars having rich porous structure in the lithium-ion batteries effectively provides reduction of “volumetric expansion effect” and “shuttle effect” on the performance (Xia et al. 2020). The highest surface area value was obtained at 500 °C pyrolysis temperatures at 50% K1 dose. Decomposition accelerates with dehydration and demethylation reactions that occur depending on the increase in the catalyst dose (Kawamoto 2017; Duan et al. 2019). Surface area of biochars mostly changes between 8 and 132 m^2^ g^−1^ (Akpasi et al. 2023). Therefore, it can be emphasized that surface area of OP catalytic biochars generated at 500 °C can be comparable with the literature values and these biochars can be used effectively in the lithium-ion batteries.

**Fig. 8:**
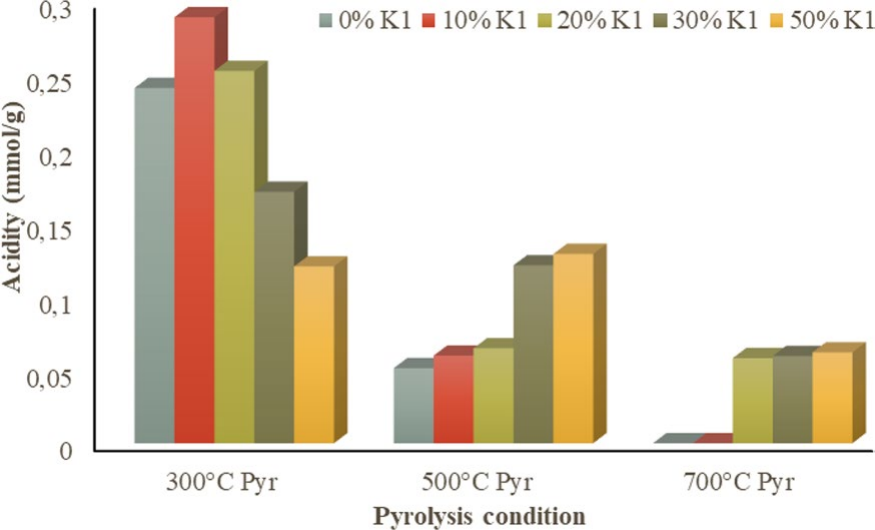
Surface acidity of pyrolysis biochars of OP and OP+K1

As for surface acidity, the biochars obtained at 300 °C pyrolysis temperature had the highest surface acidity and the surface acidity of the biochars was decreased with the increment of K1 ratio (Fig. 8). In the raw OP and all OP+K1 pyrolysis, surface acidity of biochars decreased with increasing pyrolysis temperature. Pyrolysis biochars which were obtained with the pyrolysis of raw OP and low K1 ratio (10% K1) at high temperature (700 °C) completely lost their surface acidity (Fig. 8). The alkaline properties of the biochars depend on the –COO–, –O– and the carbonate components of the biochars (Yuan et al. 2011). Moreover, surface acidity is affected from carbonization process whatever the material source. Increment in the temperature results in the increment of the total base cations and carbonates (Yuan et al. 2011; Tomczyk et al. 2020). Carbonization renders all products alkaline (pH > 7.0) at 600 °C and above. Ash content, oxygen functional groups obtained from the pyrolysis process as well as vanishment of acidic functional groups and formation of basic groups can be linked with the higher pH with increasing temperature (Al-Wabel et al. 2013; Zhao et al. 2017). Additionally, alkaline salts are also separated from organic materials due to the increased pyrolysis temperature (Ding et al. 2014; Yuan et al. 2011). Alkaline salts start separation from the organic matrix and raise the pH of the product for the temperatures above 300 °C. In contrast, cellulose and hemicelluloses decompose at about 200–300 °C and organic acids and phenolic substances, which decrease the pH of the products, are generated (Tomczyk et al. 2020). Similar to this study, Abe et al. (1998) indicated that the cellulose and hemicellulose decomposition approximately at 250–300 °C produces organic acids and phenolic substances resulting in lower pH of the product (Shinogi and Kanri 2003). Thereby, catalytic OP biochars produced in this study can be effectively used in the anaerobic digestion process due to its high buffering capacity due to the alkaline properties. It was stated that alkali biochars have great buffering capacity for overcoming acidic and/or alkaline conditions occurred in the anaerobic digestion process and the buffering capacity of biochars maximizes with the increment of earth metals (*especially Ca and Mg*) in the biochar (Zhao et al. 2021). Considering the high alkaline earth elements in the structure (Table 1), OP-K1 catalytic biochars are very appropriate for the anaerobic digestion processes due to both alkaline properties and high earth metal quantities.

#### Effect of K1 on the biochar chemical structures

**Fig. 9:**
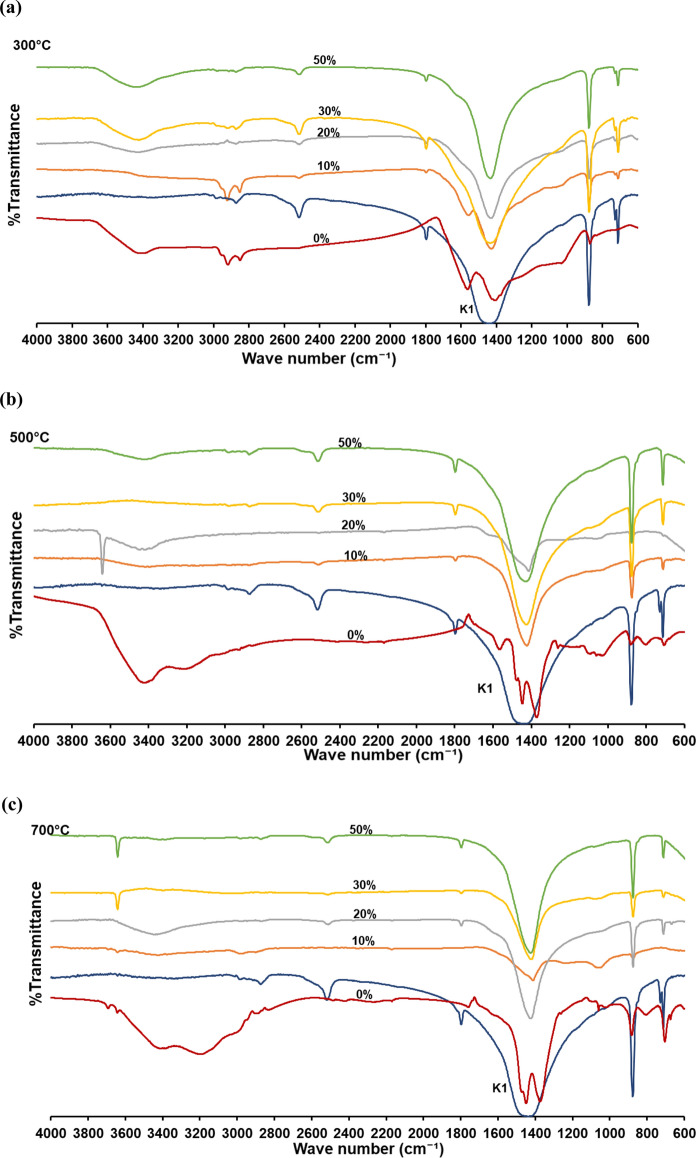
FTIR spectra of pyrolysis biochars obtained with pyrolysis of OP with different temperatures: **a** 300 °C; **b** 500 °C; **c** 700 °C

FTIR analyses were conducted for determination of the functional groups in the biochars, the binding sites and the aromatic/aliphatic state of the biochars (Fig. 9). In the FTIR spectrum of biochars produced by the pyrolysis of OP at 300 °C pyrolysis temperature without catalyst, O–H stretching around 3400 cm^−1^ and aliphatic C–H stretch bands in the range of 2918–2847 cm^−1^ were observed. Moreover, aromatic C=C at 1568 cm^−1^, aromatic ring C–H vibration at 1416 cm^−1^, lignin syringile ring at 1031 cm^−1^, and alkene C–H bond bands at 871 cm^−1^ were detected. These results matched with the other studies about pyrolysis biochar obtained from lignocellulosic biomasses in the literature (Cantrell et al. 2012; Li et al. 2013; Gulab et al. 2016). In the biochars obtained without K1 and the temperature of 300–700 °C, the intensity of the aromatic bands of around 1570 cm^−1^ decreased gradually until the pyrolysis temperature of 500 °C and disappeared at the pyrolysis temperature of 700 °C. Instead, in-plane asymmetric and symmetrical bending bands of CH_3_ formed around 1448 cm^−1^ and 1370 cm^−1^ wavenumbers at 500–700 °C pyrolysis temperatures. Aliphatic C–H stretching bands at 2918 cm^−1^ and 2850 cm^−1^ were detected only for the pyrolysis temperature of 300 °C. C–H bond of alkene compounds at 871 cm^−1^ wave number was observed for all studied temperatures (Fig. 9a). These results show that above 500 °C pyrolysis temperature, almost all the lignin in the OP was decomposed and the biochar structure consists predominantly of aliphatic carbon. Similarly, Cantrell et al. (2012) emphasized that lignin structure in the lignocellulosic biomasses degraded and aliphatic compounds increases in biochar especially at high pyrolysis temperatures.

At 300 °C pyrolysis temperature and 10% K1 dose, the bands detected for 0% K1 dose biochar continued to be seen, except the aromatic ring C–H vibration band at the same pyrolysis temperature (Fig. 9b). Although the band of OH stretch around 3400 cm^−1^ remained the same at 20% and higher K1 doses, the aromatic C=C bands at 1568 cm^−1^ and the bands of the lignin-structured syringil ring at 1031 cm^−1^ became smaller (Fig. 9c). These results show that OP decomposes more, and the biochar structure changes with increasing catalyst dose for same temperature. Although some bands belonging to aromatic and aliphatic groups were observed in the FTIR spectra of OP biochar at pyrolysis temperatures of 500 and 700 °C for 0% K1 dose, its chemical structure was similar to K1 at 10–50% K1 doses. This shows that the biochar structure was mostly formed by K1 and only catalyst bands were observed in the spectra at 20–50% K1 doses for all studied pyrolysis temperatures (Fig. 9c–e). Similarly, Irfan et al. (2019) indicated that the structure of solid residues of municipal solid waste gasification conducted with marble powder as catalyst consists of mainly CaCO_3_ and CaO.

#### Mineralogical contents of the biochars

**Fig. 10:**
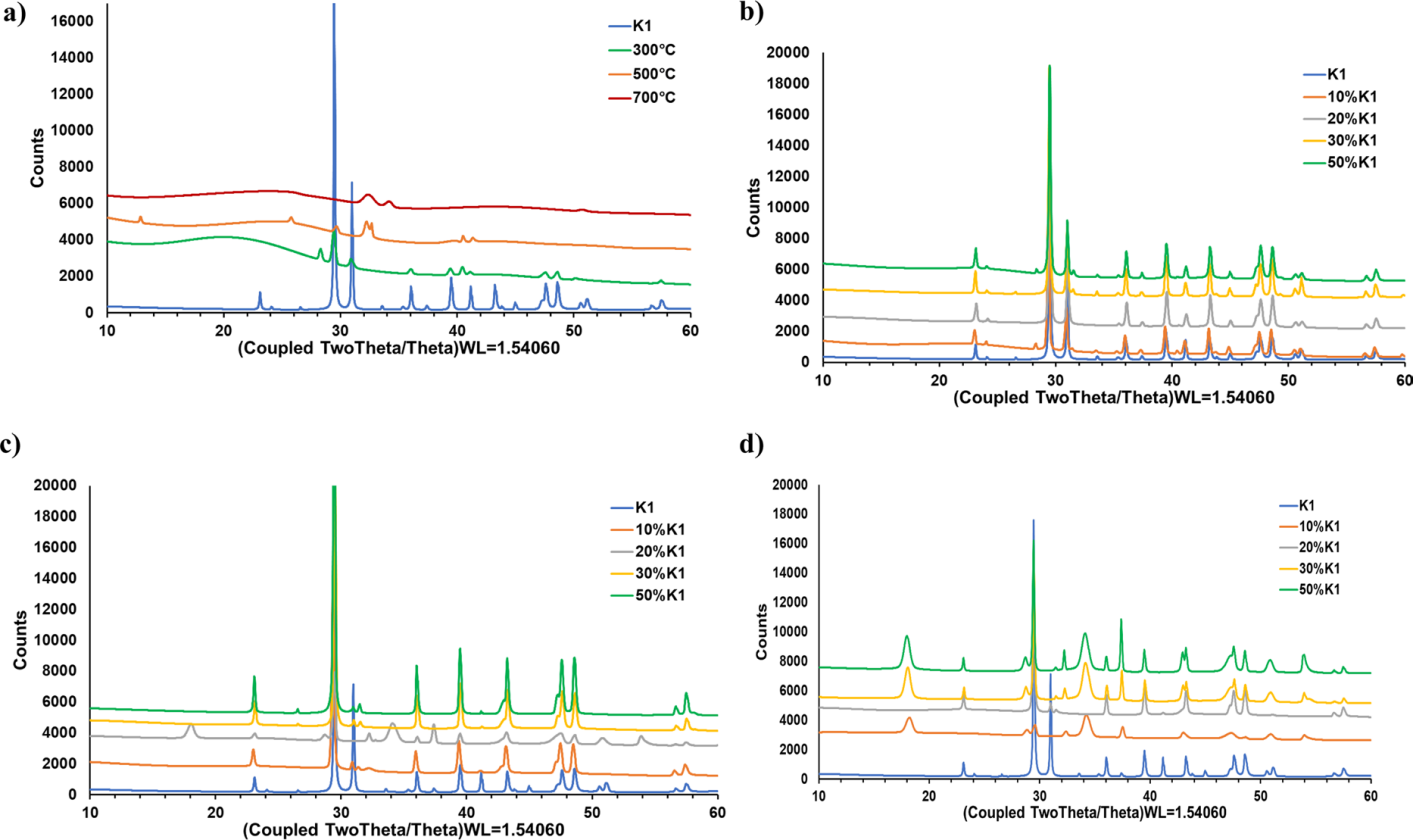
XRD patterns of OP and OP+K1 mixture biochars obtained: **a** without catalyst at 300 °C, 500 °C and 700 °C pyrolysis temperatures; **b** with 10, 20, 30, 50% K1 at 300 °C; **c** with 10, 20, 30, 50% K1 at 500 °C; **d** with 10, 20, 30, 50% K1 at 700 °C

OP includes various intrinsic inorganic elements, such as K, Ca, Fe, Mg, in addition to organic compounds and these elements remain into the pyrolysis biochar after pyrolysis since they do not decompose during the thermochemical process. Furthermore, these intrinsic inorganic elements provide to OP self-catalyzing property as indicated in literature (Dinc and Yel 2018). K1 catalyst used in this study also includes same inorganic elements, so biochars obtained from OP-K1 mixture pyrolysis became rich in terms of inorganic contents which shows that this can provide new different alternative usage area for produced biochars. On the other hand, it is necessary to proof that K1 remain as its after the pyrolysis process to be able to use catalyst. For all these reasons, XRD analyses were conducted to evaluate the mineralogical contents of the biochars and XRD patterns of uncatalyzed biochars were presented in Fig. 10a and the possible components of each pattern determined as a result of matching the XRD patterns with the library search were presented in Table 5.

**Table 5:** Variation of possible mineralogical components in OP+K1 biochars with pyrolysis temperature and K1 dose

Pyrolysis temperature	Possible mineralogical components	K1 dose										
		0%	10%		20%		30%		50%		K1	
300 °C Pyr	CaCO_3_ calcite		**√**				**√**		**√**		**√**	
	CaMg(CO_3_)_2_ dolomite		**√**		**√**		**√**		**√**		**√**	
	(Mg_0.03_Ca_0.97_)(CO_3_) magnesium calcium carbonate	**√**			**√**							
	CaMg_0.77_Fe_0.23_(CO_3_)_2_ Ca–magnesium iron carbonate	**√**										
500 °C Pyr	CaCO_3_ calcite		**√**				**√**		**√**		**√**	
	CaMg(CO_3_)_2_ dolomite										**√**	
	(Mg_0.03_Ca_0.97_)(CO_3_) calcite, magnesium syn				**√**							
	Ca(OH)_2_ calcium hydroxide				**√**							
	CaO calcium oxide				**√**							
700 °C Pyr	CaCO_3_ calcite				**√**		**√**		**√**		**√**	
	CaMg(CO_3_)_2_ dolomite				**√**						**√**	
	CaO calcium oxide						**√**		**√**			
	Ca(OH)_2_ calcium hydroxide		**√**				**√**		**√**			

Kristiani et al. (2015) emphasized that the peaks at 2θ = 13.3–17.3°; 18.4–25.6° and 32.06–36.26° regions indicate cellulose. The diffraction peaks shown around 2θ = 14° and 36° in the XRD pattern of OP pyrolysis biochar generated at 300 °C may belong to the crystal plane for cellulose types I and II (Fig. 10a). In addition, the peak in the wide region around 2θ = 20.5° is a characteristic peak belonging to the cellulose type I and II crystal planes (Sunday Samuel and Mathew Adefusika 2019). Lignin is also an amorphous polymer, and its characteristic peak will coincide with the cellulose peak around 2θ = 20–21° depending on the biomass type (Lu et al. 2012; Singh et al. 2015a). Undegraded lignin in the 300 °C pyrolysis biochars can be observed since the complete degradation of lignin will continue until very high temperatures. Peaks for anhydrite (sulphate group of minerals) at 2θ = 29.5°; for CaCO_3_ at 43.2° and 50.2°; for silvite (KCl) at 48.3° and for halite (NaCl) minerals at 57.4° were observed (Nanda et al. 2013). Cellulose peaks were not observed in pyrolysis biochars obtained at pyrolysis temperatures of 500 °C and above. This finding is also compatible with the degradation information of cellulose given in the literature (Yang et al. 2007; Kabakci and Aydemir 2014). A very small peak of graphitic carbon was observed at 2θ = 44° in pyrolysis biochars at 500 °C and it became more apparent at 700 °C pyrolysis biochars. Therefore, it can be emphasized that the amount of amorphous carbon decreases and the amount of graphitic carbon increases by pyrolysis temperature increment (Hasan Khan Tushar et al. 2012). In addition, new peaks formed at 2θ = 26° in pyrolysis biochars at 500 and 700 °C also indicate graphitic carbon (Singh et al. 2015b). In the literature, a wide band in the range of 20–30° indicates the presence of a weak crystal structure and a carbon-rich phase in these samples (Zhang et al. 2018).

On the XRD patterns of the biochars obtained as a result of pyrolysis at 300 °C and low catalyst ratio (10%), there was a peak around 28° (2θ) corresponding to the cellulosic content of the OP in the crystal structure (Altun 2018) (Fig. 10b). The catalyst structure remains stable with the increment of pyrolysis temperature as specified in the FTIR findings of the biochars. Therefore, it is expected that a large amount of K1 will be found in the structures of biochars with the increment of K1 in the pyrolysis process. CaCO_3_ and CaMg(CO_3_)_2_ peaks belonging K1 were observed between 20–25° (2θ) and 30–50° (2θ) in the XRD pattern of biochars (Fig. 10c). CaMg(CO_3_)_2_ peaks observed at approximately 31°–41°–45°–51° (2θ) in the catalyst XRD pattern were not observed in pyrolysis biochars above 500 °C since MgCO_3_ started to decompose at temperatures of approximately 500 °C. Especially at 30% and 50% K1 ratio, Ca(OH)_2_ peaks around 18°–29°–34°–47°–51° (2θ) and CaO peaks (32°–37.5°–54° (2θ)) were observed by virtue of the degredation of CaCO_3_ when the pyrolysis temperature rises to 700 °C (Fig. 10d; Table 5). Consequently, the pyrolysis biochars of OP+K1 mixtures contain mainly CaCO_3_, CaMg(CO_3_)_2_ and (Mg_0.03_Ca_0.97_)(CO_3_) (Table 5). This result is critical for usage of catalytic biochars of OP in the new green approaches such as direct carbon solid oxide fuel cell (DC-SOFC). Xie et al. (2021) indicated that alkaline metal and alkaline earth metal elements in the biochar have positive effect on the electrochemical reactions by supplying more CO resulting in improvement of DC-SOFC performance. OP-K1 catalytic biochars include high quantity of alkali metal elements since both raw OP and K1 also include these elements at high amounts as indicated before. Therefore, it can be specified that catalytic biochars generated in this study may be good option for DC-SOFC.

#### Elemental analysis

**Fig. 11:**
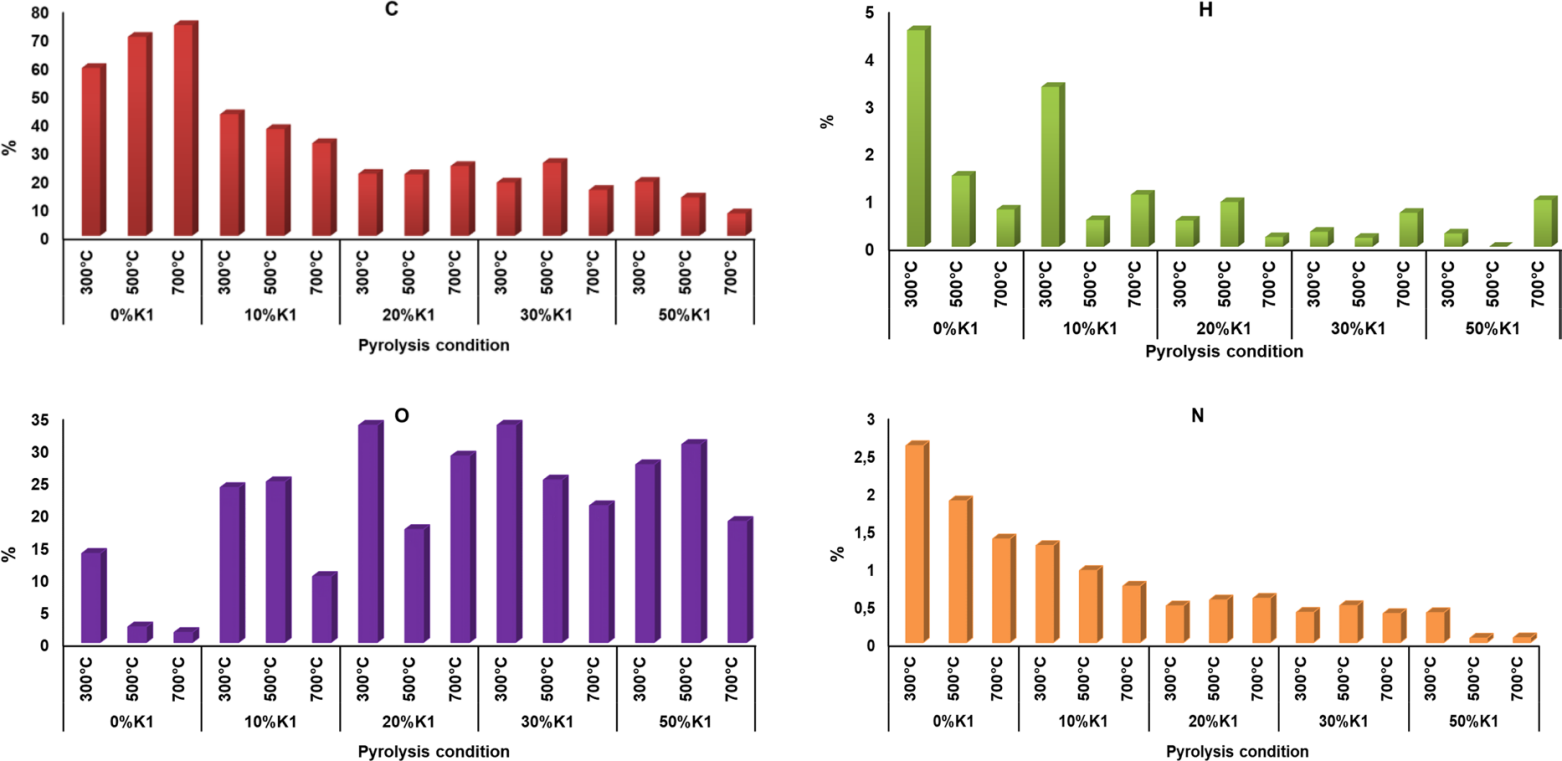
Elemental composition of OP+K1 pyrolysis biochars obtained under different pyrolysis conditions

**Fig. 12:**
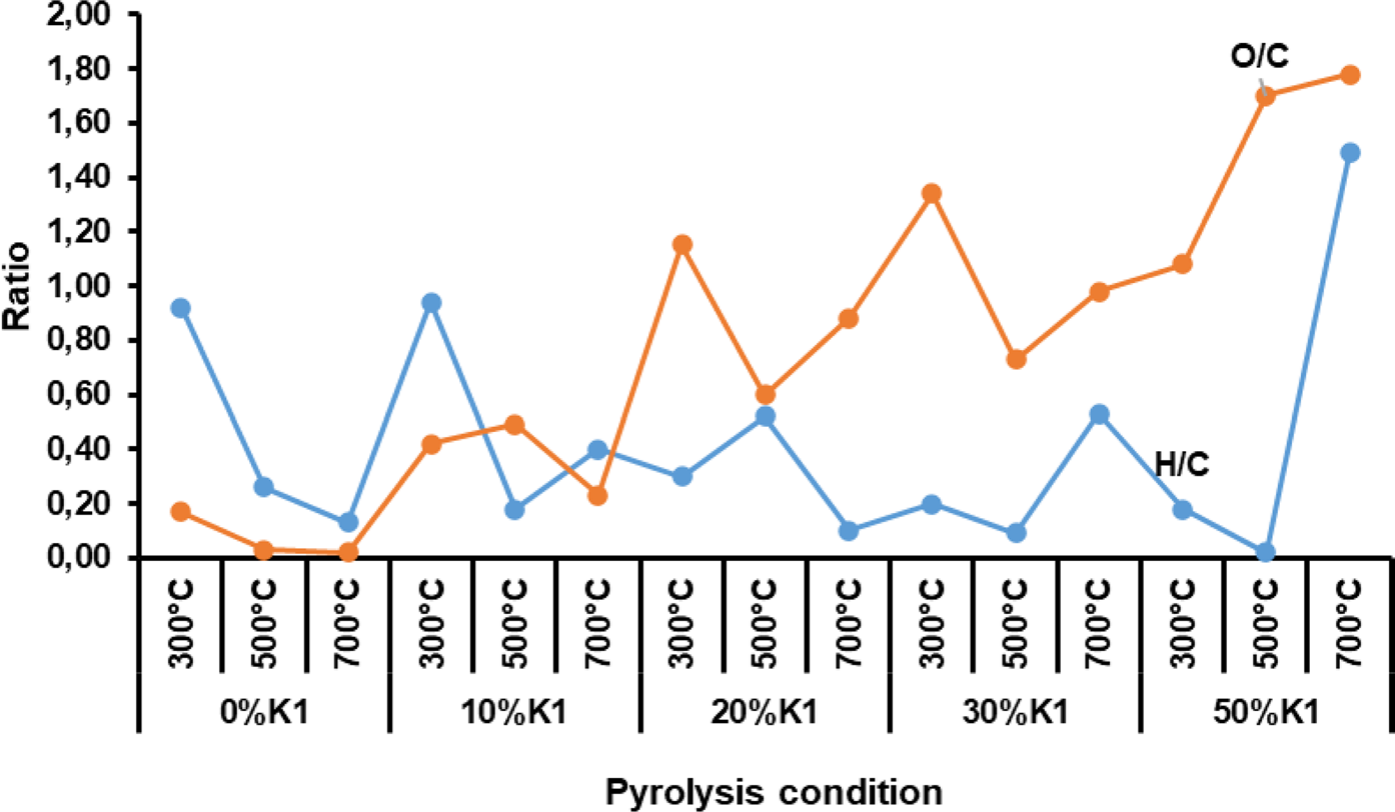
H/C atomic ratio of pyrolysis biochars obtained at different catalyst doses and pyrolysis temperatures (Ratios of H/C and O/C were expressed on a molar basis; H/C ratio of unpyrolyzed OP = 1.6)

The elemental composition of the pyrolysis biochars obtained at different catalyst doses and temperatures of OP and OP+K1 mixtures was presented in Fig. 11. O percentages given in elemental compositions were obtained by subtracting C, N, H, S, ash, and moisture values from the total percentage. The H/C and O/C molar ratios calculated from the elemental compositions of the pyrolysis biochars were presented in Fig. 12.

Elemental composition of raw OP was 26.7% C, 3.7% H, 1.5% N. Therefore, it can be emphasized that C and O exist at high percentage in OP, but quantity of H and N elements are low. Additionally, raw OP did not include S element. Therefore, both OP catalytic and noncatalytic pyrolysis biochars did not include S element and this property is very critical for the usage biochar in thermal treatments. The low S content will reduce the air pollution potential in thermal treatments which means that usage of biochars presented in this study make contribution to the processes of the green technology. Biochars of lignocellulosic biomasses and coals mostly have high content of sulfur. The sulfur content in the coal changes between 0.2 and 5% by dry weight. Therefore, this sulfur content is converted to hydrogen sulfide (H_2_S) during the energy production process from coals and this can cause health and environmental problems (URL 1).

C percentage of pyrolysis biochars obtained from raw OP and OP+K1 mixtures were much higher than raw OP. While the H/C ratio of raw OP was 1.6, this ratio was much lower for all pyrolysis biochars obtained except for a few pyrolysis conditions (Fig. 12). For biomass, the H/C ratio generally varies between 1.4 and 1.8 (Hu and Gholizadeh 2019) and the value obtained for raw OP also supports the literature. The low H/C ratio in pyrolysis biochar shows that biochars are products with a more aromatic structure than raw biomass. However, since the pyrolysis biochars in this study are not only biomass biochar but also contain the inorganic catalyst, the elemental analysis findings also include C and O elements from the catalyst structure.

In OP pyrolysis biochars obtained without use of a catalyst (0%), while the percentage of C in the pyrolysis biochars increased from 59 to 74; the percentage of H decreased from 4.5 to 0.78; and O from 13.8 to 1.5 by rising the pyrolysis temperature from 300 to 700 °C. Low H content indicates conversion of hydrogen into bio-oil and non-condensable gas phases (Zhang et al. 2017; Dönmez et al. 2022). The increase in gas product volume with increasing pyrolysis temperature supports this finding (Fig. 5). The increment of C percentage with the increase of pyrolysis temperature is compatible with the literature. For instance, the C content of orange pomace biochar increased from 56.8 to 68.1% with the increase of pyrolysis temperature (Tag et al. 2016). Similarly, biochar carbon ratio rose from 27 to 35% with increasing pyrolysis temperature (Cantrell et al. 2012). Carbon content increment by pyrolysis temperature increment is arise from high polymerization and denser carbon structure in the biochar (Tomczyk et al. 2020).

**Fig. 13:**
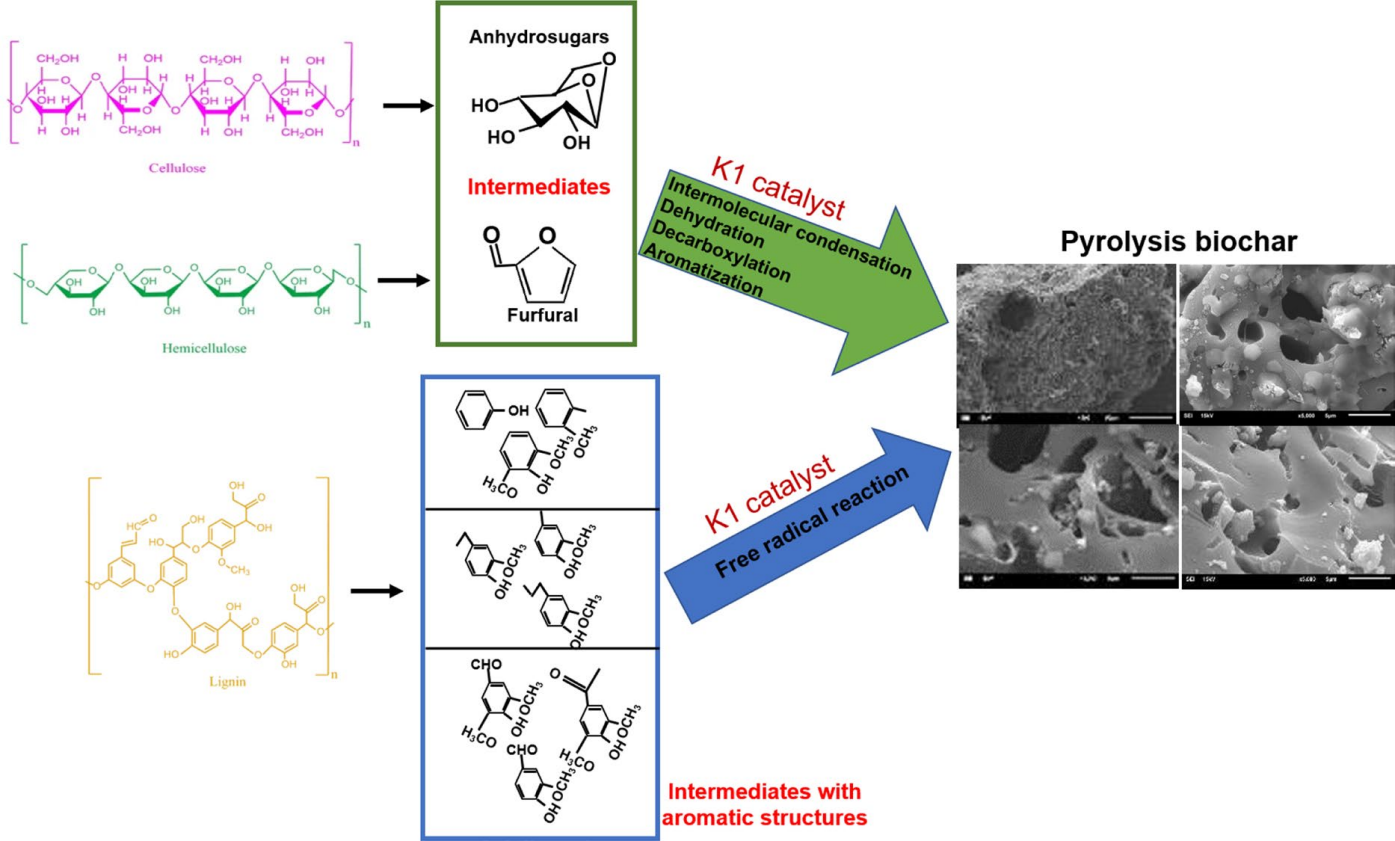
Main intermediate structures formed during OP degradation and reactions to form biochar with the K1 catalysts (Prepared with the evaluation of this study results by utilization of literature studies) (Hu and Gholizadeh 2019; Li et al. 2022)

When the elemental analyzes of the pyrolysis biochars of OP with different doses of K1 are compared with the pyrolysis biochars without catalyst, the C, H and N percentages mostly decrease with the increasing catalyst dose, while the O percentage increases (Fig. 11). Lower C percentages in the biochars obtained by the pyrolysis of K1 and OP together indicates that the C structures were converted into liquid and gaseous products by increasing the decomposition of cellulose, hemicellulose and lignin structure in OP biomass during the pyrolysis of K1. Main intermediate structures during OP degradation and reactions to form biochar with the K1 catalysts were presented in Fig. 13. While intermolecular condensation, dehydration, decarboxylation, and aromatization are the main reaction type for converting intermediates of cellulose and hemicellulose compounds to the biochar, free radical reaction is predominated during the lignin decomposition (Fig. 13). It can be emphasized that K1 had a catalytic effect on these reactions during the OP pyrolysis since Ca, Mg, Al, hydroxides, and carbonates exist in K1 structure. For instance, Gulab et al. (2016) indicated that CaCO_3_ favors aromatization reaction and results increment in aromatic structures during the *Eichhornia crassipes* biomass pyrolysis. In other study, it was stated that alkali and alkaline earth metals (such as Ca, Mg) in zeolite structure promotes catalytic efficiency by effecting aromatization, dehydration, carboxylation reactions during biomass pyrolysis (Wang et al. 2022).

The difference in O percentages in catalytic and non-catalytic pyrolysis biochars can be expressed that K1 increases the degradation of lignin in the OP structure during pyrolysis. In catalytic pyrolysis, lignin can first be broken down into oxygenates such as phenols. Subsequently, polycondensation of lignin and repolymerisation of phenols can occur, resulting in biochar formation (Hu and Gholizadeh 2019) (Fig. 13). Catalyst pores can fill up with the produced oxygen compounds and so oxygen ratio in the biochars may rise. In addition, CaO and Ca(OH)_2_ compounds observed in the biochar structure together with the degradation of CaCO_3_ observed in the catalyzed pyrolysis biochars also affect the amount of oxygen in the structure. While the H/C and O/C ratios decreased by increment of pyrolysis temperature in most of the pyrolysis biochars, the highest O/C ratios were mostly obtained at low pyrolysis temperatures (Fig. 12). This shows that the pyrolysis biochars produced at high temperatures have lower oxygen content.

#### Thermal properties of the biochars

**Fig. 14:**
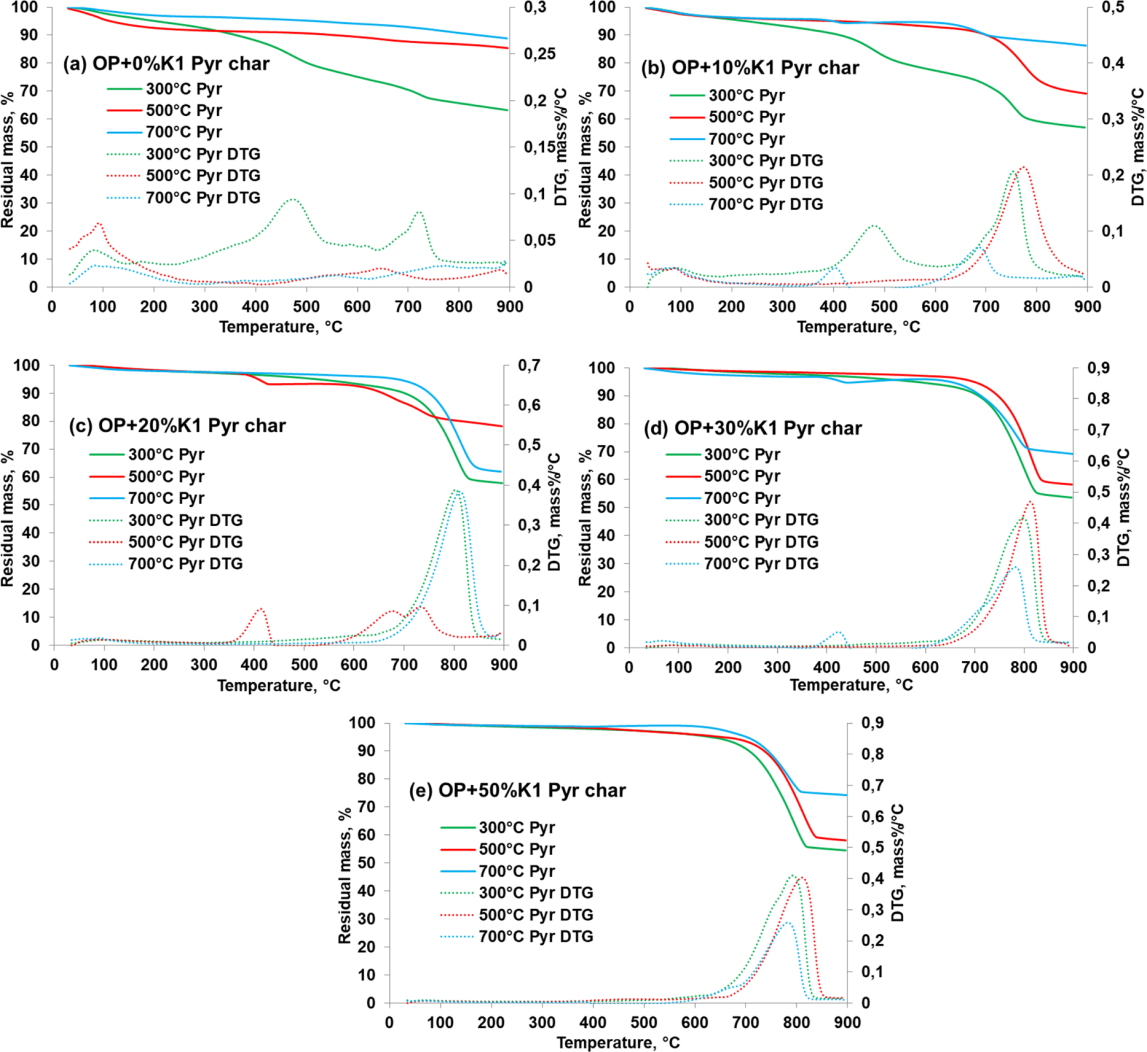
TGA-DTG thermograms of pyrolysis biochars obtained with pyrolysis of OP with different K1 doses: **a** 0% K1; **b** 10% K1; **c** 20% K1; **d** 30% K1; **e** 50% K1

TGA and DTG curves of pyrolysis biochars obtained with different K1 doses and pyrolysis temperatures of OP waste were presented in Fig. 14. According to Fig. 11, while the highest mass loss for pyrolysis biochars of OP (%0 K1) was observed at pyrolysis biochars obtained at 300 °C, the least mass loss was obtained in the pyrolysis biochars at 700 °C. There were more undegraded compounds in the pyrolysis biochars obtained at 300 °C as compared to other pyrolysis biochars since some of the compounds in the OP structure did not decompose at this pyrolysis temperature. FTIR graphs of OP pyrolysis biochars also support these results. According to the FTIR spectra of OP pyrolysis biochars (Fig. 9), while there are aromatic carbons in the structure of pyrolysis biochars obtained at 300 °C, it was observed that the structure of pyrolysis biochars above 500 °C pyrolysis temperature consisted mainly of aliphatic carbon and almost all of the lignin in OP was decomposed. Similarly, in the DTG curves of OP biochars obtained without catalyst, although it was observed the peak due to the degradation of the raw OP by the release of light molecular weight volatile substances in its structure at 157–357 °C pyrolysis temperature, the second peak at about 370–500 °C was not observed. The temperature range in which the second peak is observed coincides with the decomposition temperatures of both cellulose and lignin, and these groups in the OP structure did not decompose at 300 °C and remained in the pyrolysis biochar. At the end of 900 °C, the remained weight of uncatalyzed OP biochars in the TGA graph increased as the temperature increased. This can be explained by the fact that the organic structures are less in the pyrolysis biochars obtained at high temperatures and the remaining inorganic structure does not decompose too much.

The thermal degradation trend of pyrolysis biochars obtained with pyrolysis of OP+K1 differ from biochars obtained from OP pyrolysis alone. The residuals of the pyrolysis biochars of OP+K1 mixtures at 500 °C and 700 °C temperatures at the end of 900 °C TGA temperature were higher than the OP biochars without K1 (Fig. 14). This can be connected to the decomposition of K1 in the pyrolysis biochars of OP+K1 mixtures at high temperatures. TGA thermograms of OP biochars obtained at 300 °C pyrolysis temperature and 10% K1 dose showed similarity to OP pyrolysis biochar without catalyst at the same temperature (Fig. 14). The same similarity was also observed in the FTIR spectrum of these biochars (Fig. 9). TGA graphs of 500 °C and 700 °C pyrolysis biochars were similar to the K1 TGA curve; therefore, it can be emphasized that the effect of K1 is effective at temperatures above 300 °C for 10% and above doses. Moreover, it can be stated that OP does not complete its decomposition at 300 °C pyrolysis temperature, either alone or in combination with K1.

When the thermograms of the pyrolysis biochars obtained at different temperatures for the same K1 doses are examined (Fig. 14), it can be stated that the peak temperature values at which the maximum degradation is seen in the DTG graphs have the lowest value for the biochars obtained at 700 °C compared to the other pyrolysis biochars. This can be expressed by that some of the K1 in the mixture decomposes during pyrolysis in the experiments carried out at 700 °C and this degradation reaction affects the pyrolysis process. Unlike other biochars, some peaks were observed in the DTG graphs of 700 °C pyrolysis temperature-10% and 30% K1 doses and 500 °C-20% K1 dose (Fig. 14). These peaks can be attributed to the degradation of Ca(OH)_2_. As indicated in XRD analysis of pyrolysis biochars, CaCO_3_ (calcium carbonate-Calcite), CaMg(CO_3_)_2_ (Dolomite) and (Mg_0.03_Ca_0.97_)(CO_3_) (calcite) were found as the main components. However, apart from the main components, Ca(OH))_2_ and CaO compounds were also observed in the biochar structures due to the formation of CO_2_ in the environment with the decomposition of OP, the two-stage decomposition of K1 in 700 °C pyrolysis and the decomposition of CaCO_3_ with increasing pyrolysis temperature (500–700 °C). The decomposition temperature of Ca(OH)_2_ in pyrolysis biochar takes place in the range of 350–550 °C (Kim and Olek 2012).

Addition to TGA-DTG graphs, the thermal resistance values of the biochar products obtained from the pyrolysis runs were evaluated and given in Table 6. Two-stage degradation was observed for the pyrolysis biochars of 300 °C 0% and 10% K1 conditions. Initial degradation temperatures (DT_*1_) for these pyrolysis biochars were found as 310 °C and 321 °C, respectively. The secondary decomposition temperatures (DT_*2_) were between 503.7 and 655.3 °C (Table 6). As mentioned before, the two-stage degradation observed in these pyrolysis biochars is due to the non-degraded cellulose and lignin structures in the biochar.

**Table 6:** Thermogravimetric findings of pyrolysis biochars of OP+K1 mixtures

Sample			Thermal resistance values					Residual at 900 °C (%)
Type	K1 dosage	Pyrolysis temperature (°C)	DT_*1_ (°C)	DT_*2_ (°C)	T_5_ (°C)	T_10_ (°C)	T_50_ (°C)	
K1	–	–	613.4	–	713.4	751.0	–	54.9
OP – K1-5 °C min^−1^ Pyrolysis biochar	0%	300	310.0	503.7	203.9	361.6	> 900	63.2
		500	550.9	–	115.1	557.0	> 900	85.4
		700	446.4	–	520.0	839.1	> 900	88.9
	10%	300	321.0	507.1	224.6	413.0	> 900	57.0
		500	588	–	407.6	701.4	> 900	69.1
		700	382.0	655.3	299.4	702.9	> 900	86.3
	20%	300	567.8	–	524.4	701.1	> 900	57.9
		500	386.7	638.6	408.4	657.3	> 900	78.2
		700	665.2	–	685.9	750.7	> 900	62.0
	30%	300	575.1	–	577.9	707.0	> 900	53.6
		500	602.7	–	697.5	746.5	> 900	58.3
		700	439.2	610.0	431.1	712.0	> 900	69.2
	50%	300	552.8	–	632.3	706.8	> 900	54.5
		500	520.6	–	654.1	736.4	> 900	58.1
		700	642.4	–	702.0	743.3	> 900	74.3

*T*_*5, 10, 50*_ temperatures at which 5%, 10% and 50% decomposition occurs

According to Table 6, the temperature required for the 5% and 10% degradation of the pyrolysis biochars obtained as a result of the pyrolysis of OP alone generally increased as the pyrolysis temperature increases. From this point of view, it is concluded that OP biochars become more difficult to thermally decompose as the pyrolysis temperature at which it is fractioned increases. According to SEM images (Fig. 6), as the pyrolysis temperature increases, the porous structure increases, resulting in less degradable structure compared to low pyrolysis temperatures, which also supports this result.

T_5_ and T_10_ values of OP biochars obtained at 0% K1 changed between 203.9–596.8 °C and 361–839 °C, respectively as the pyrolysis temperature increased. T_50_ values of all OP biochars were found as higher than 900 °C. The residual amounts of OP pyrolysis biochars obtained at 900 °C were higher than raw OP (21.4%). As the pyrolysis temperature increased, the degradation of biochars obtained from OP pyrolysis without catalyst slowed down and increased from 63.21 to 88.86% when temperature increase from 300 to 700 °C, respectively. It can be stated that irregularities at other pyrolysis temperature between 300 and 700 °C are due to the effect of the impurities in the raw OP waste on the biochar product after pyrolysis. DT_*2_ values of OP-K1 mixture pyrolysis biochars varied between 503 and 655 °C. The high temperatures of DT_*2_ values in organic waste pyrolysis using mineral catalysts also support these results (Merdun and Laougé 2021). T_5_, T_10_ values and the complex organic compounds in their structures increased when pyrolysis temperatures and K1 dose increased. According to the SEM images, as the pyrolysis catalyst dose increased, the porous structure of the pyrolysis biochars increased (Fig. 6), indicating that the biochar proceeded to a difficult degradation stage. T_50_ values for all pyrolysis temperatures and catalyst doses of OP+K1 biochars were above 900 °C. The ambient temperature must reach temperatures higher than 550–600 °C to degrade biochar products obtained from pyrolysis of cellulosic wastes and high-dose mineral catalysts by 50% (Bu et al. 2021). Moreover, it can be said that the reason for the T_50_ values of OP+K1 pyrolysis biochars to be above 900 °C is that the CaO in the K1 structure makes the biochar structure more thermally resistant due to the organic composition of OP (Irfan et al. 2019).

Studies showed that the pomace, with its low ash content and high calorific value, creates an important renewable energy source potential as an alternative to coal (Altun 2018). Sustainable primary and secondary energy production from biomass is extremely important considering the decreasing fossil fuel reserves. The dry OP average calorific value is 3500–4000 kcal kg^−1^, which is comparable to wood (3890 kcal kg^−1^) (Dinc and Yel 2020). For this purpose, the heat values of the biochar products obtained from OP alone (without catalyst) and with K1 pyrolysis experiments were compared (Table 7). The OP samples used in this study contain a large amount of moisture in their original form. While wet OP sample had a calorific value of 84.3 cal g^−1^, calorific value of dried OP was 3010 cal g^−1^. In the pyrolysis experiments of this study, OP samples were used in their original form taken from the facility without drying. Therefore, the heating values of wet OP+K1 mixtures before pyrolysis were found as low and ranged from 5 to 11 cal g^−1^ (Table 7). Most of the hemicellulose, cellulose and volatile substances in the OP decompose at between 177 and 380 °C, which is called the active site in the TGA graph. Therefore, no significant effect of pyrolysis temperature was observed for OP samples without K1 in the studied temperature range, and the heating values of noncatalytic OP pyrolysis biochars were measured in the range of 5500–6288 kcal kg^−1^ (Table 7).

**Table 7:** Heat values of OP+K1 pyrolysis biochar

Study findings						Literature (Jahirul et al. 2012; Akdag et al. 2018)	
Pyrolysis temperature	Heat value, cal g^−1^					Alternatives used as fuel	Heat value cal g^−1^
	0% K1	10% K1	20% K1	30% K1	50% K1		
Raw OP	84.3	6.9	7.0	5.3	11.3	Anaerobic sludge	1900–3500
300 °C Pyr	5500.1	3708.3	2052.1	1495.2	647.1	Sewage sludge	2700–2900
500 °C Pyr	6288.4	3378.8	2480.6	1054.0	666.9	Peat	4100
700 °C Pyr	5546.8	3203.3	1881.2	1293.9	1089.1	Lignite	3800
						Coke	6200

**Fig. 15:**
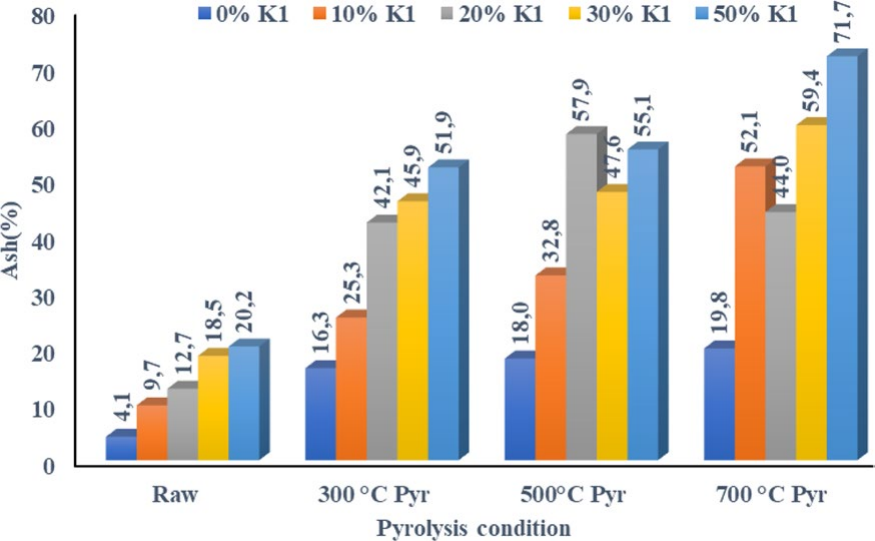
Ash values of OP+K1 pyrolysis biochar

Although it was observed a decrement in the calorific values of pyrolysis biochars of OP+K1 mixtures at all studied pyrolysis temperatures because of the low calorific value of K1, heating values of OP-K1 biochars are still comparable with the literature values up to 20% K1 dosage (Table 7). Moreover, heat values of some OP-K1 biochars are higher than the coal types such as lignite. The increment of the K1 dose in the OP+K1 mixture also increased thermal resistance as indicated in TGA analysis. Therefore, it can be indicated that TGA and heat value finding of pyrolysis biochars compromise to each other. Since OP thermal decomposition takes place at low temperature, the calorific value of biochar did not significantly change with increasing pyrolysis temperature in all catalyst ratios (Table 7). Another important point in the usage of produced biochars at energy generation process is increment of ash quantities by catalyst dosage and pyrolysis temperature increment (Fig. 15). Raw OP without K1 contains about 4% ash and the ash content in pre-pyrolysis OP+K1 mixtures ranges from 5.5 to 24% (Fig. 15). As the pyrolysis temperature increases, the carbon and ash content of biochar increases (Chen et al. 2008; Fuertes et al. 2010). Rafiq et al. (2016) reported that increasing pyrolysis temperature caused an increment in ash content of 5.7–18.7%. The increase in ash content is due to the increased concentration of inorganic components and organic matter combustion residues (Cao and Harris 2010; Chen et al. 2014; Zhao et al. 2017; Tomczyk et al. 2020). Moreover, the increased ash content is the result of a decrement of the content of other elements during pyrolysis. While the elements, such as nitrogen, carbon, hydrogen, oxygen, and sulfur, volatilize during heating, the inorganic salts comprising the ash do not evaporate, so the ash content increases (Shinogi and Kanri 2003). Ash end products after energy production process should be taken into consideration and be found solutions for these residuals as emphasized in the literature (Chen et al. 2008; Fuertes et al. 2010). Therefore, if studied catalytic biochars are used for energy production purposes, ash products will consist of high quantities of inorganic substances, which means that they can be used proposed areas indicated before or can be used as catalyst over and over in pyrolysis process.

All in all, it can be emphasized that presented catalytic biochar can be used at many different and new areas; however, using as a catalyst again in pyrolysis processes can be stated as the most logical approach for achieving utilization more than one way. The remaining part can be evaluated in other alternative areas mentioned above after decreasing their catalyst features when the produced catalytic biochars are used repeatedly in the system as a catalyst.

## Conclusion

In this study, two different types of wastes, olive pomace (OP) and marble sludge (K1) produced by physicochemical treatment of marble processing effluent were pyrolysed together and the effects of K1 on the potential green applications of OP pyrolysis biochar and pyrolysis product yields were investigated in detail. K1 showed an important catalytic effect on the OP pyrolysis even at low dosage and the following conclusions were drawn:

- As a result of the pyrolysis of OP and K1 mixtures, the increment in the temperature for the same K1 dose mostly reduced the amount of pyrolysis biochar and the lowest biochar amounts were obtained at 700 °C. This decrease shows that K1 accelerates the decomposition reactions of OP and decreases the biochar product yields depending on the pyrolysis temperature.
- Raw OP pyrolysis biochars obtained at 300 °C pyrolysis temperature looked like a smooth, oily adherent mass and the biochar surface became a little rougher and porous even at the 10% K1 dose, which also supports the catalytic effect of K1 in the decomposition of OP.
- The presence of K1 with high thermal resistance increased the thermal resistance of the biochars of OP+K1 mixtures.
- OP biochars obtained at 300 °C pyrolysis temperature has the highest surface acidity, and the biochar surface acidity decreased with increasing K1 ratio and pyrolysis temperature. Alkaline properties of produced biochars provides to the biochars high buffering capacity for anaerobic digestion processes. Moreover, it can be indicated that these biochars can have a potential usage for lithium-ion batteries, and direct carbon solid oxide fuel cell (DC-SOFC) due to the alkaline characteristics, but further electrochemical property test and detailed study on this application should be performed.
- The high mass loss at uncatalyzed OP pyrolysis biochars at 300 °C was attributed to the decomposition of aromatic hydrocarbon compounds to form phenol. Above 500 °C pyrolysis temperature, almost all of the lignin in OP was decomposed, the biochar structure was predominantly aliphatic carbon and aromatic structure decreases with K1.
- The crystalline cellulosic content of pomace was found in the biochar products obtained as a result of the pyrolysis process at 10% catalyst ratios and 300 °C pyrolysis temperature. In OP+K1 pyrolysis biochar structures, CaCO_3_, CaMg(CO_3_)_2_ and (Mg_0.03_Ca_0.97_)(CO_3_) were observed. Moreover, Ca(OH)_2_ and CaO was found due to the decomposition of CaCO_3_ with increasing pyrolysis temperature. Therefore, it can be specified that catalytic biochars generated in this study has potential to be a good option for direct carbon solid oxide fuel cell (DC-SOFC) due to both alkaline properties and high quantity of inorganic content (especially Ca and Mg). This is another recommended application for further investigation.
- The calorific value of char did not change significantly with increasing pyrolysis temperature at all catalyst ratios as thermal decomposition in OP occurred at low temperature and K1 increased the thermal strength.
- If studied catalytic biochars are used for energy production purposes, ash products will consist of high quantities of inorganic substances, which means that they can be used proposed areas indicated before or can be used as catalyst over and over in pyrolysis process.
- An increase in ash content of biochars was observed with increasing K1 ratio and pyrolysis temperature. This was connected to be due to the presence of K1 as well as inorganic components and organic matter degradation residues.
- While the percentage of C, H and N mostly decreased, the percentage of O increased with increasing dose and pyrolysis temperature in the presence of K1. The difference in O percentages in catalytic and non-catalytic pyrolysis biochars shows that the C structures in the biochar are transformed into liquid and gaseous products by increasing the degradation of lignin in the OP structure of K1 during pyrolysis. Therefore, it can be indicated that composition and valuable compounds in the pyrolysis products should be investigated more detail.

These findings showed that K1 has an important effect on both pyrolysis yields and characteristics and biochars having different qualities were produced by using K1 at different pyrolysis conditions. One of the main valuable features of the study is that it suggests simultaneous disposal of two different types of wastes by converting both of them into valuable new feedstocks. Moreover, with this study, besides the development of an economical and environmentally friendly approach in the catalytic pyrolysis of OP, new potential alternative green application areas were presented according to the characteristic features of the produced biochars. This suggested approach can help establishment of industrial symbiotic relationships between these two industries.

## Data Availability

The datasets used and/or analyzed during the current study are available from the corresponding author on reasonable request.
